# PPE64 is a mycomembrane channel protein that functions in heme iron uptake and moonlights in biofilm formation in *Mycobacterium tuberculosis*

**DOI:** 10.1128/mbio.03281-25

**Published:** 2025-12-11

**Authors:** Padam Singh, Charles B. Kaufman, Lisa Whitworth, Reed M. Stubbendieck, Randy Morgenstein, Karen L. Wozniak, Avishek Mitra

**Affiliations:** 1 Department of Microbiology and Molecular Genetics, Oklahoma State University 7618, Stillwater, Oklahoma, USA; 2 OSU Microscopy Laboratory, Oklahoma State University 7618, Stillwater, Oklahoma, USA; NYU Langone Health, New York, New York, USA

**Keywords:** *Mycobacterium tuberculosis*, iron acquisition, heme, biofilm, outer membrane, mycomembrane channel protein, PPE

## Abstract

*Mycobacterium tuberculosis* (*Mtb*) is the leading cause of human deaths by an infectious agent. To survive in the human host, *Mtb* must acquire essential iron nutrients from the host and evade the immune response. In diderm bacteria like *Mtb*, outer membrane channel transporter proteins are fundamental for nutrient acquisition and immune evasion. Recently, we demonstrated that the *Mtb* outer mycomembrane PPE64 is a channel protein, providing the first direct evidence of channel-forming capability by a protein of the PPE (proline-proline-glutamate motif) family, which are found exclusively in mycobacteria. Here, we demonstrate that the PPE64 channel protein is specifically required for the uptake of heme, which is the largest source of iron in the human host. Furthermore, PPE64 plays a crucial role in biofilm formation, and this function is not dependent on the iron source in the medium. The moonlighting function of PPE64 is reflected in its two distinct oligomeric states, where the higher-order oligomer specifically forms water-filled channels in membranes and binds heme. The discrete roles of PPE64 are also important in an *ex vivo* setting, as its absence significantly impairs *Mtb* growth within human monocytes and alveolar macrophages. Thus, our study reveals that the PPE64 channel protein plays multiple roles in *Mtb* physiology and establishes it as an important factor in *Mtb* virulence.

**IMPORTANCE:**

The success of any invading bacteria to survive within the host is dictated by their ability to acquire nutrients and overcome the host immune response. Bacterial cell surface proteins play critical roles in these processes at the host-pathogen interface. Here, we show that the PPE64 mycomembrane channel protein is required for heme iron acquisition and biofilm formation, which are fundamental processes that are of great significance to *Mycobacterium tuberculosis* (*Mtb*) survival within the host. These discrete functions of PPE64 are dictated by the culturing environment and are important for *Mtb* growth within human macrophages. These observations support an emerging theme in the *Mtb* field that the PPE protein family functions in trafficking molecules across the outer mycomembrane and has far-reaching implications for understanding of *Mtb* physiology.

## INTRODUCTION

*Mycobacterium tuberculosis* (*Mtb*) kills ~1.3 million people yearly and has surpassed HIV/AIDS to become the leading cause of human deaths by an infectious agent (1). The ability of *Mtb* to overcome nutrient limitations in the host and evade the immune response is the principal reason that makes it such a successful pathogen. Following *Mtb* entry into the human lung, resident macrophages phagocytose *Mtb* and activate the nutritional immunity response to sequester metals (2, 3) like iron, zinc, manganese, and magnesium, which are required for vital biological functions (4). In this intracellular environment, *Mtb* is constantly battling to acquire metal nutrients from the host for its own survival. However, *Mtb* also spends a key part of its life cycle as extracellular bacilli within the granuloma (5). Numerous studies have shown that extracellular *Mtb* grow within biofilms and have linked biofilm growth to immune evasion and antibiotic treatment tolerance (6–10). These observations are not surprising given that bacteria primarily exist within biofilms in nature and bacterial biofilms are major drivers of chronic infections and antibiotic tolerance (11–13). Thus, understanding the principles of *Mtb* nutrient acquisition and biofilm formation is of great relevance, as this knowledge will allow us to develop targeted strategies to inhibit vital processes in *Mtb*.

Diderm bacteria (like *Mtb* [14, 15]) use canonical β-barrel outer membrane proteins (OMPs) to traffic molecules across their outer membrane. Expectedly, β-barrel OMPs are central to the survival and pathogenicity of gram-negative bacteria because they function in fundamental biological processes, such as uptake of essential metal nutrients (2, 16), outer membrane stabilization (17), motility (18), biofilm formation (19–21), and antibiotic resistance (22). Many studies (23, 24) have employed bioinformatic approaches to identify putative *Mtb* β-barrel OMPs and some *Mtb* mycomembrane proteins (25–28) with channel-forming capability and solute transport have been identified. However, we still do not have direct structural evidence of *Mtb* β-barrel OMPs. In recent years, a growing body of evidence has shown that some proteins of the proline-proline-glutamate (PPE) motif family function in transporting molecules across the *Mtb* mycomembrane. For example, PPE36/PPE62 (29, 30), PPE37 (31), and PPE4 (32) function in iron acquisition, PPE20 (33) and PPE31 (34) function in calcium and magnesium transport, respectively, and PPE51 (34–38) functions in the trafficking of various nutrient molecules. Notably, PPE proteins are exclusively found in mycobacteria (39, 40). These observations led to a hypothesis that some proteins of the PPE family can function as channel proteins to transport molecules across the mycomembrane.

In our most recent study, we provided the first direct evidence for this emerging hypothesis by demonstrating that PPE64 is a mycomembrane protein that forms channels in lipid membranes (41). In our current study, we demonstrate that the PPE64 channel protein is essential for heme (Hm) iron uptake. This is of great significance because the majority (>75%) of the iron in our body is stored as Hm within hemoglobin (42) in blood, making Hm the largest reservoir of circulating iron in the human host (43, 44). Thus, PPE64 plays an important role in *Mtb* iron acquisition. PPE64 also has broader roles in *Mtb* physiology influencing cell size and biofilm formation. Finally, we demonstrate that these distinct roles of PPE64 are necessary for *Mtb* growth within human macrophages, establishing PPE64 as an important factor for *Mtb* virulence.

## MATERIALS AND METHODS

### Bacterial strains, growth media, and preparation of iron-free medium

Wild-type (wt) *Mtb* H37Rv and its derivative strains were grown in Middlebrook liquid 7H9 or solid 7H10 medium supplemented with 10% ADS (8.5 g/L NaCl, 20 g/L dextrose, and 50 g/L bovine albumin fraction V), 0.5% glycerol, 0.2% casamino acids (CAAs), and 0.02% tyloxapol. This fully supplemented medium is referred to as complete 7H9 medium from hereon. Biofilm assays for *Mtb* strains were performed in standard Sauton’s medium containing 0.5 g/L K_2_HPO_4_, 0.5 g/L MgSO_4_, 4.0 g/L L-asparagine, 2 g/L citric acid, 6% glycerol, and 0.05 g/L ferric ammonium citrate with a final pH of 7.2. *Escherichia coli* DH5α was grown in either LB medium containing appropriate antibiotics at 37°C with shaking at 180 rpm. The following antibiotics were used when required: ampicillin (Amp) at 100 μg/mL for *E. coli*; kanamycin (Kan) at 30 μg/mL for mycobacteria and 50 μg/mL for *E. coli*; and hygromycin (Hyg) at 200 μg/mL for *E. coli* and 50 μg/mL for mycobacteria.

Both 7H9 and Sauton’s contain ~150 µM ferric citrate. For iron-free 7H9 or iron-free Sauton’s, all components except ferric citrate were dissolved in Millipore water in acid-washed beakers to prepare the base iron-free media. Lyophilized albumin was added to iron-free 7H9 base medium to prepare the base iron-free albumin-7H9 medium. Medium was then filter sterilized through a 0.2 μM filter. Freshly made ferric citrate or hemin solution was added to either base medium to prepare the specific iron-containing medium. Hemin solutions at 20 mM concentration were prepared in DMSO. The iron chelators 2,2-dipyridyl (DIP) and deferoxamine (DFO) were prepared in DMSO and added to freshly made hemin medium.

### Targeted gene deletion in *Mtb* and construction of gene expression vectors

To construct mutants, 1,000 bp of left (L) and 1,000 bp right (R) flanking sequences of the target gene were amplified using corresponding primer pairs LF/SpeI-LR/SwaI and RF/PacI-RR/NsiI (Table S3), respectively, and cloned into pML2424 to construct gene deletion vectors (Table S2). The deletion vectors were then transformed into *Mtb*. Transformants were selected at 37°C on 7H10 Hyg and visually validated through the presence of both GFP and RFP fluorescence. Liquid culture of transformant was then plated on 7H10 Hyg containing 2% sucrose at 40°C for selection of double crossovers. Putative double crossovers were visually analyzed for the presence of only GFP, and gene deletion was validated by PCR. To excise the *loxP*-flanked *gfp*^2+^*_m_-hyg* cassette, pML2714-expressing Cre recombinase was transformed into marked mutants, and unmarked mutants were selected on 7H10 Kan at 37°C. Putative unmarked mutants were first visually validated through the absence of GFP fluorescence and then through PCR (Fig. S1) (validation primers (V/F – V/R), Table S3) and loss of growth on hygromycin. All validated mutants were designated an OAL# (Table S1) for identification. The orf of *ppe64* containing its native promoter and ribosome-binding site was cloned into pDM101 or pML1335 vectors to generate the episomal (pDM103) or integrative (pDM106) mycobacterial expression vectors, respectively. pOAL301, expression vector for purification of PPE64, was constructed in our previous study (41). The orf of *ppsA* containing its native ribosome-binding site was cloned into pDM101 to generate the episomal pOAL410 mycobacterial expression vector.

### Growth experiments for determining iron utilization

**Fig 1:**
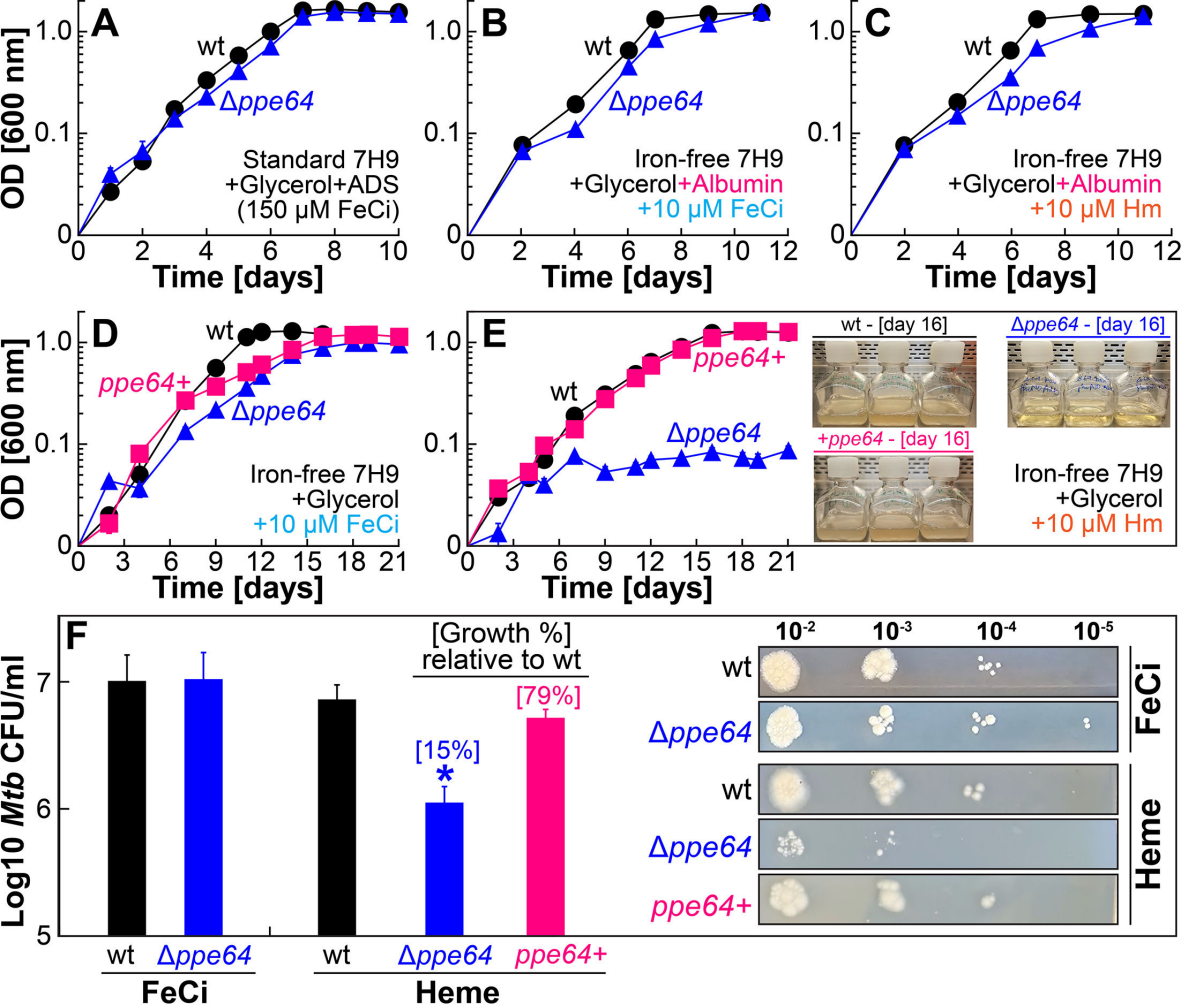
PPE64 is critical for Hm iron utilization by the non-albumin Hm uptake pathway. (**A** ) Growth of wt (black) and Δ*ppe64* (blue) strains in standard liquid 7H9 medium which contains 150 µM ferric citrate (FeCi) and has been supplemented with 1% glycerol, 10% ADS, and 0.02% tyloxapol. (**B and C**) Growth of strains in iron-free liquid 7H9 medium containing 1% glycerol, 0.02% tyloxapol, and 75 µM albumin, which has been supplemented with either 10 µM FeCi (**B**) or 10 µM Hm (**C**).(**D and E**) Growth of wt, Δ*ppe64,* and complement (pink) strains in iron-free liquid 7H9 medium containing 1% glycerol and 0.02% tyloxapol, which has been supplemented with either 10 µM FeCi (**D**) or 10 µM Hm (**E**). Inset in E shows triplicate cultures of growth experiment at day 16. (**F**) Growth of strains on self-made iron-free solid 7H10 agar plates supplemented with 1% glycerol, 10% ADS, and 0.02% tyloxapol containing either 10 µM FeCi or 10 µM Hm. Single-cell suspension of iron-depleted strains was normalized to an OD_600_ of 0.05, then serially diluted, and 5 µL of each dilution was spotted on agar plates. FeCi and Hm agar plates were imaged on day 21 and 35, respectively, and CFU counts were determined from either 10^−3^ or 10^−4^ dilution. The right panel shows a representative image of plates used to determine CFU counts. Uncropped plate images are shown in Fig. S2. All Hm medium contains 20 µM of the iron chelator DIP to prevent utilization of trace iron. All error bars represent the standard error of mean (SEM) values of biological triplicates. In many cases, error bars are smaller than the marker data points. Asterisk denotes Δ*ppe64* is significantly different from wt. Statistical significance was determined by Tukey’s HSD following an F-test (*P* < 0.05). The source data file is provided.

**Fig 2:**
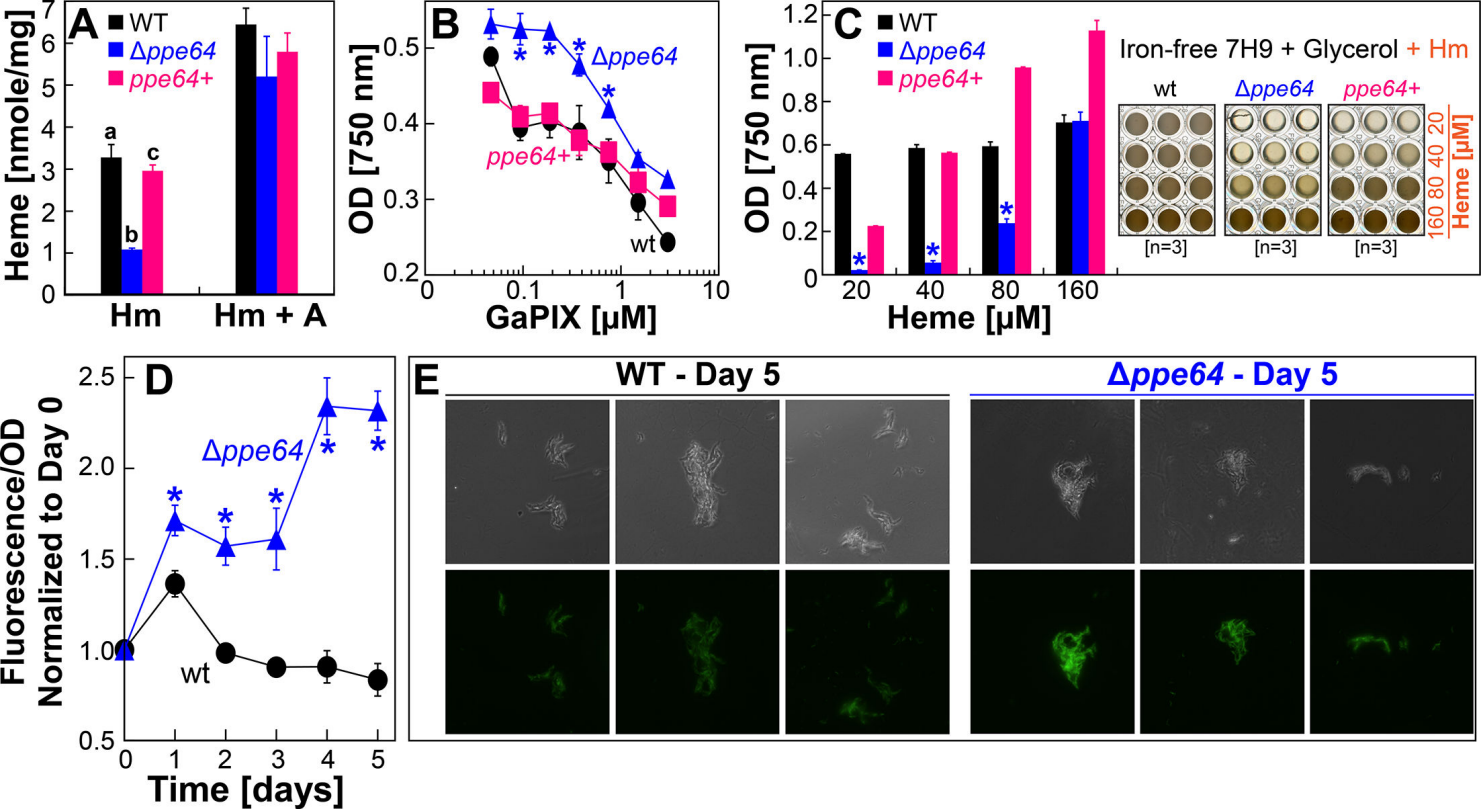
PPE64 influences Hm uptake in *Mtb*. (**A** ) Quantification of Hm levels in wt (black), Δ*ppe64* (blue), and complement (pink) strains by hemochromogen assay. Iron-depleted strains were inoculated into iron-free liquid 7H9 medium supplemented with either 10 µM Hm or 10 µM Hm and albumin (Hm + A). Hm levels in strains were quantified after 48 h by the pyridine hemochromogen assay and then normalized to the total protein amount in whole-cell lysates. (**B**) Growth of strains in iron-free liquid 7H9 medium containing varying amounts of the toxic Hm analog gallium-protoporphyrin IX (GaPIX). To allow for the background level of growth, 1.0 µM FeCi was included as an iron source. Growth was determined by measuring endpoint OD 750 on day 7. (**C**) Growth of strains in iron-free liquid 7H9 medium containing increasing levels of Hm. Strains were grown in 96-well plates, and growth was determined by measuring endpoint OD 750 on day 35. The right panel shows images of 96-well plates. (**D**) Determination of Hm levels using a cytoplasmic Hm biosensor. Green fluorescence (GF) from cytoplasmic Hm biosensor in strains grown in iron-free liquid 7H9 supplemented with 10 µM Hm. GF from Hm biosensor was monitored at 24-h intervals, normalized to OD 750, and is reported relative to day 0. (**E**) Hm biosensor fluorescence in strains examined by microscopy. GF was monitored at the same exposure levels for both strains. Raw image files are provided. All error bars represent the SEM of biological triplicates. For A, plots with different lowercase letters indicate statistically significant differences from each other. Asterisk denotes Δ*ppe64* is significantly different from wt. Statistical significance was determined by Tukey’s HSD following an F-test (*P* < 0.05). Source data file is provided.

Unless specified, all liquid cultures were grown in sealed square PETG bottles with shaking at 120 rpm, and all incubation was done at 37°C and all liquid and solid growth medium experiments were performed in triplicate. Strains were first grown to the mid-exponential phase in complete 7H9, then washed in sterile PBS containing tyloxapol, and then iron-depleted for five generations in iron-free 7H9 medium containing ADS, glycerol, CAA, and tyloxapol. This iron-depletion protocol was strictly performed before all growth experiments. Iron-depleted cells were passed through a 5.0 μM filter to obtain a single-cell suspension, which was then used to inoculate iron-free liquid 7H9 or plate on iron-free solid 7H10 agar containing specific iron sources as mentioned in the main text. For plating on solid agar plates, single-cell suspension was prepared at an optical density (OD)_600_ of 0.05, which was serially diluted, and 5 µL of each dilution (specified in the figure) was spotted on agar plates. Bovine serum albumin was added to the medium to a final concentration of 0.5% wt/vol (75 µM) for albumin growth experiments. Unless specified, all Hm medium (liquid and solid) in our study contained 20 µM of the iron chelator DIP. For growth curve experiments (Fig. 1A through E, 5B and 6A), strains were inoculated in 30 mL of medium at an initial OD_600_ of 0.01. For endpoint growth experiments (Fig. 2B and C, 6B), strains were inoculated in a final volume of 200 µL in 96-well plates at an initial OD_600_ of 0.001 in wells containing varying concentrations of Hm. OD of liquid cultures was measured using a BioTek Synergy plate reader. All experiments were performed with a minimum of three biological replicates.

### Assessing Hm levels by hemochromogen assay, Hm biosensor, and fluorescence microscopy

Strains were grown to the mid-log phase in complete 7H9, iron-depleted, and then inoculated at an OD_600_ of 1.0 into 30 mL of iron-free 7H9 containing either 10 µM Hm or 10 µM Hm and 75 µM albumin. Strains were harvested after 48 h, and Hm level was determined by hemochromogen assay (45). Briefly, cells were first resuspended in 30 mL of 1× PBS, lysed by sonication, then clarified by low-speed centrifugation at 1,500 × *g* for 5 min to collect the whole-cell lysate (WCL), and total protein was quantified by BCA assay. To extract Hm, 500 µL of WCL was added to a cuvette into which 0.2 M NaOH, 40% pyridine, and 200 µM potassium ferricyanide were added in 500 µL. Released Hm was then reduced by adding 10 µL of 0.5 M DTT, and absorbance was measured at 557 nm. Hm was quantitatively determined using the extinction coefficient 32.4 mM^−1^ cm^−1^ and then normalized to total protein amount WCL.

The *hs1*-M7A Hm biosensor expression vector pYUB1874 (46) was transformed in wt, and Δ*ppe64* and transformant were selected on 7H10 Kan agar plates. Iron-depleted strains were inoculated at an OD_600_ of 1.0 in triplicate into 10 mL of albumin-free iron-free 7H9 containing 10 µM Hm, and cells were harvested for temporal analysis. Biosensor green fluorescence (GF) from green fluorescent protein (eGFP) was monitored by excitation at 480 nm and emission at 510 nm, normalized to OD of cultures, and reported relative to day 1. OD and fluorescence were measured using a BioTek Synergy plate reader. GF from Hm biosensor strains was also analyzed by microscopy.

### Assessing susceptibility to GaPIX toxicity

Strains were first grown to the mid-log phase in complete 7H9 and then iron-depleted as usual. Iron-depleted cells were passed through a 5.0 μM filter to obtain a single-cell suspension, which was then inoculated in a final volume of 200 µL in 96-well plates at an initial OD_600_ of 0.001 in 7H9 medium containing 1 µM ferric citrate and varying concentrations of GaPIX. OD of cultures was measured using a BioTek Synergy plate reader on day 7. All experiments were performed with a minimum of three biological replicates.

### Electron microscopy analysis

For transmission electron microscopy (TEM), cells were fixed in 2% glutaraldehyde in 0.1 M Na Cacodylate for a minimum of 2 h. After three washes (180 mM sucrose in 60 mM Na Cacodylate), cells were fixed in 1% OsO4 (aqueous) for 1 h, followed by three washes (180 mM sucrose in 60 mM Na Cacodylate) and then dehydration in increasing concentrations of ethanol. Cells were washed 3× in propylene oxide as a transitional solvent and then infiltrated with a 1:1 mixture of propylene oxide and EMbed 812 resin. After the removal of propylene oxide, cells were embedded in 100% EMbed812. Thin sections (80–90 nm thick) were stained with uranyl acetate and lead citrate and then viewed with JEOL JEM2100 TEM. For scanning electron microscopy (SEM), cells were fixed in 2% glutaraldehyde in 0.1 M Na Cacodylate for a minimum of 2 h. After three washes (180 mM sucrose in 60 mM Na Cacodylate), cells were placed on poly-L-lysine-coated coverslips. Cells on coverslips were then fixed in 1% OsO4 (aqueous) for 1 h, followed by three washes (180 mM sucrose in 60 mM Na Cacodylate) and then dehydration in increasing concentrations of ethanol. Samples were washed 2× in hexamethyldisilazane for 5 min each and allowed to air dry. Samples were then coated with Au/Pd and imaged in an FEI Quanta 600 FEG SEM.

### Ethidium bromide accumulation assay

Strains were first grown to the log phase in 30 mL of complete 7H9 medium and then filtered through a 5.0 μM filter to obtain a single-cell suspension. Cells were then harvested by low-speed centrifugation at 1,500 × *g* for 10 min and resuspended to a final OD_600_ of 1.0 in uptake buffer (76 mM (NH_4_)_2_SO_4_, 0.5 M KH_2_PO_4_, 1 mM MgSO_4_, 0.4% glucose, and 0.05% Tween-80). For all strains, 100 μL of cells was added in triplicate in a 96-well plate, and ethidium bromide (EtBr) was then added to a final concentration of 20 µM. Fluorescence was measured by excitation at 530 nm and emission at 590 nm at 1-min interval for 60 min.

### Lipid extraction and thin layer chromatography

Lipid extractions were performed following established protocols (47–50). For all lipid extractions, all strains were first grown to an OD_600_ of 1.0 in 50 mL of complete 7H9 medium. For extraction of total apolar lipids, cells were harvested by centrifugation, and the pellet was resuspended by adding 2 mL of methanol-0.3% NaCl (10:1, vol/vol) solution and 1 mL of petroleum ether and then mixed on an end-over-end rotor for 30 min. The sample was then centrifuged at 4,000 × *g* for 5 min, and the upper layer containing phthiocerol dimycocerosates (PDIMs) was collected in a fresh tube. Another 1 mL of petroleum ether was added to the bottom layer, mixed, centrifuged again, and the upper layer was collected. Upper layer fractions were combined, dried, and then samples were spotted onto thin layer chromatography (TLC) plates and resolved in petroleum ether-diethyl ether (90:10, vol/vol). Strains were also labeled with ^14^C-propionate, and PDIMs were detected by autoradiography for 72 h using a Typhoon Phosphor Screen. For the extraction of surface lipids, cell pellets were resuspended in 5 mL of hexanes and mixed on an end-over-end rotor for 5 min. Samples were centrifuged at 3,000 × *g* for 5 min, hexane-extracted lipids were collected in a fresh tube. An equal amount of chloroform-methanol (2:1, vol/vol) was added. Samples were dried, resuspended in chloroform-methanol (2:1, vol/vol), and then spotted onto TLC plates. TAG was resolved by TLC in toluene-acetone (80:20, vol/vol). TMM, TDM, and free mycolic acids (FM) were resolved by TLC in chloroform-methanol-water (90:10:1, vol/vol/vol). All TLC plates were dipped in 10% molybdophosphoric acid in ethanol, and lipids were then visualized by charring plates.

### Biofilm growth assays

Strains were first grown to the mid-log phase in complete 7H9 and then iron-depleted as usual. Iron-depleted cells were passed through a 5.0 μM filter to obtain a single-cell suspension, which was then inoculated in 24-well plates at a final OD_600_ of 0.01 in iron-free Sauton’s medium containing either 10 µM ferric citrate or 10 µM Hm. Plates were sealed with parafilm, wrapped in aluminum foil, and then incubated static at 37°C. After 5 weeks, media was carefully aspirated using a 25G needle, 1 mL of 1% crystal violet (CV) was added to each well and incubated at 37°C for 10 min. CV was then removed from the wells, biofilm was washed gently 2× with PBS, and 1 mL of 95% ethanol was added to the wells. CV was extracted for 10 min at 37°C, and biofilm mass was then quantified by measuring absorbance at 590 nm. For planktonic growth experiments in Sauton’s medium (Fig. 5C), all protocols were performed exactly as described for 7H9 medium, except tyloxapol was added to a final concentration of 0.02% in Sauton’s medium.

### Van10-P vancomycin susceptibility assay

Strains were first grown to the mid-log phase in complete 7H9 supplemented with 0.1 mM propionate and passed through a 5.0 μM filter to obtain a single-cell suspension. Cells were then inoculated in a final volume of 200 µL in 96-well plates at a final OD_600_ of 0.005 in complete 7H9 medium containing 10 µg/mL vancomycin. Cells were incubated for 10 days, and then viability was measured by Alamar Blue assay. The growth percent of strains in vancomycin was determined relative to the growth of strains in the absence of vancomycin.

### Purification and refolding of PPE64 and analysis of channel activity in lipid bilayers

The *ppe64* expression vector pOAL301 was used to purify recombinant PPE64 from *E. coli* BL21. PPE64 was purified under denaturing conditions, isolated by nickel chromatography, and refolded in different detergents exactly as we did in our previous study (41). Bilayer experiments were performed using very similar instrumentation and methods as described by Zakharian et al. (51). Synthetic diphytanoyl phosphatidylcholine (DphPC, Avanti Polar Lipids, Birmingham, AL) was used to form planar lipid bilayers. Lipids were solubilized in *n*-Decane at 20 mg/mL, and a glass capillary tube was used to paint a bilayer in an aperture of 200 µM diameter in a Delrin cup (Warner Instruments, Hamden, CT). The bilayer was painted between an aqueous solution of 1 M KCl, 10 mM HEPES, pH 7.1, and capacitance was registered in the range of 66–100 pF. Approximately ~40 ng of purified detergent refolded PPE64 in 1–2 µL volume was added to the *cis* compartment, and channel-forming activity was recorded at 50 mV applied potential. Current trace was recorded with a patch clamp amplifier (BC-535 Bilayer Clamp, Warner Instruments). The *trans* and *cis* solutions were connected to the headstage point with Ag-AgCl electrodes. Currents were low-pass filtered at 10 kHz and then digitized through an analog-to-digital converter (Digidata 1550B, Molecular Devices, San Jose, CA). Data filtering was done at 100 Hz through an 8-pole Bessel Filter (Lpf-8, Warner Instruments) and digitized at 1 kHz using pClamp11 software (Molecular Devices). Single-channel conductance events were identified automatically using Clampfit 11 from five independent membrane recordings.

### Absorption spectroscopy for detecting Hm binding

Fresh solutions of hemin were prepared in Tris buffer as described previously (52). An equimolar amount of Hm was added to 10 μM protein and incubated at room temperature for 5 min. For difference absorption spectroscopy, Hm binding was monitored using a Bio-Tek Synergy HT plate reader by subtracting the free Hm spectra from the protein-incubated Hm spectra.

### Cell culture of THP-1 and generation of alveolar macrophage-like cells from human peripheral blood mononuclear cells

THP-1 cells (ATCC# TIB-202) were cultured in base RPMI media (containing 1% L-glutamine and 0.1% BME, 0.005% MEM essential and non-essential amino acids) supplemented with 10% FBS. This medium is referred to as complete RPMI, which was filter-sterilized through a 0.22 µm filter. THP-1 cells were incubated in T-75 flasks at 37°C in 5% CO_2_ and were split according to the manufacturer’s instructions. Prior to use, cells were harvested and quantified using a hemacytometer with trypan blue exclusion dye.

Alveolar macrophage-like cells (AMLs) were differentiated from human peripheral blood mononuclear cells (PBMCs) as previously described (53). Briefly, frozen de-identified human PBMCs (Charles River Laboratories) were thawed and seeded at 6 × 10^6^ cells/well in filter-sterilized RPMI 1640 media with 10% pooled human serum in a six-well ultra-low attachment tissue culture plate. 100 µg/mL Infasurf (ONY Biotech), 10 ng/mL GM-CSF, 5 ng/mL TGF-β, and 5 ng/mL IL-10 (Peprotech) were added at days 0, 2, and 4. On days 2 and 4, 1 mL of fresh media was added to each well after aspirating 1 mL of spent media. On day 6, cells were dissociated with Versene cell dissociation reagent (Gibco) and gentle shaking on an orbital shaker. The cell purity was verified by flow cytometry using HLA-DR and CD11c and evaluated on a NovoCyte 3000 and analyzed using NovoExpress Software (Agilent). Cell concentration was quantified with trypan exclusion dye in a hemacytometer.

### Infection assays with THP-1 and AMLs

The day before infection with *Mtb* strains, THP-1 cells were seeded in 96-well plates at 10^5^ cells/well in 100 µL RPMI containing 50 nM PMA (phorbol 12-myristate 13-acetate), and cells were then differentiated into macrophages overnight. AMLs were similarly seeded into 96-well plates at 10^5^ cells/well in 100 µL RPMI. AML RPMI medium contained Infasurf, GM-CSF, and TGF-β to sustain the AML phenotype. The following day, macrophage monolayers in all wells were washed 2× with sterile PBS, 100 µL of infection medium (RPMI supplemented with 10% non-heat-inactivated normal human serum) was added to all wells, and then *Mtb* cells in 20 µL sterile PBS were added to all wells at an MOI of 10 and an MOI of 2 for THP-1 and AML infection, respectively. All *Mtb* strains were first grown to the mid-log phase in complete 7H9, and cells were then passed through a 25G needle to obtain a single-cell suspension for infecting macrophages. Infected monolayers were incubated at 37°C in 5% CO_2_ for 3 h and then washed 2× with sterile PBS to remove extracellular bacteria, and then 100 µL of RPMI containing 10 µg/mL gentamicin was added to all wells. Medium was then removed from wells at indicated timepoints, macrophages were lysed with 100 µL 0.05% SDS, and *Mtb* was then enumerated by plating on 7H10 agar plates.

### Identification of suppressor mutations

We performed whole-genome sequencing of wt, Δ*ppe64*, and Δ*ppe64*_sup_ at Molecular Biology and Cytometry Research at the OUHSC core facility using NextSeq 2000. We processed the raw reads using fastp v0.23.2 (54) with default settings. We then used snippy v4.6.0 (55) with default settings to identify mutations in these strains, compared to the reference H37Rv genome (GenBank: AL123456.3). To identify probable suppressor mutations, we directly compared the list of single-nucleotide polymorphisms in the Δ*ppe64*_sup_ and parental Δ*ppe64* strain.

## RESULTS

### PPE64 is critical for Hm utilization by the non-albumin Hm uptake pathway

In our previous study (41), we discovered that *Mtb* H37Rv significantly upregulates the expression of the gene *ppe64* in the presence of Hm iron. Subsequently, we characterized PPE64 in binding assays, membrane localization, and channel activity experiments with specific controls to demonstrate that PPE64 binds Hm and most consequentially that PPE64 is localized in the outer mycomembrane and forms water-filled channels (41). These observations suggested that the PPE64 channel protein may play a role in Hm iron utilization in *Mtb*. To determine whether PPE64 is required for *Mtb* Hm utilization, we used homologous recombination to construct an unmarked Δ*ppe64* deletion strain (Fig. S1 and Table S1). We first monitored the growth of Δ*ppe64* in standard 7H9 liquid medium, which contains 150 µM ferric citrate (FeCi), supplemented with glycerol and ADS (albumin, dextrose, salt). Under these conditions, wt and Δ*ppe64* exhibit nearly identical growth (Fig. 1A), ensuring that Δ*ppe64* does not have any generalized growth defect. As Mitra et al. (29) have shown that *Mtb* has two Hm uptake pathways, termed albumin and non-albumin, we next examined whether PPE64 is required for either of these pathways. We monitored the growth of Δ*ppe64* to utilize albumin-Hm by growing strains in iron-free 7H9 supplemented with glycerol and albumin containing either 10 µM FeCi (Fig. 1B) or 10 µM Hm (Fig. 1C). Growth of wt and Δ*ppe64* is nearly identical under these conditions, demonstrating that PPE64 is not required for Hm utilization by the albumin pathway. We next monitored the growth of Δ*ppe64* to utilize non-albumin Hm by growing strains in iron-free 7H9 supplemented with glycerol containing either 10 µM FeCi or 10 µM Hm. In the absence of albumin, Δ*ppe64* exhibits only a marginal growth delay in FeCi (Fig. 1D), but deletion of *ppe64* significantly reduces the growth of *Mtb* in the presence of Hm after day 6 (Fig. 1E). The initial growth for 6 days as observed for Δ*ppe64* in Hm medium has also been observed in other *Mtb* Hm utilization mutants in our previous studies (29, 30, 41), and this background growth was attributed to either utilization of residual iron from the medium or residual internal iron reserves in the cells of the *Mtb* strains. Regardless, the growth of Δ*ppe64* is fully restored to wt levels through complementation from the episomal expression vector pDM103 (Table S2), which expresses *ppe64* using its native promoter. The Hm growth phenotype of Δ*ppe64* is also recapitulated by plating for CFU on self-made iron-free 7H10 solid agar plates containing either 10 µM FeCi or 10 µM Hm. Since growth on agar plates is significantly slower than in liquid media and because our complement strain OAL154 (Table S1) requires hygromycin, for agar plate assays, we used wt and Δ*ppe64* strains expressing the empty expression vector pDM101 (Table S2) to control for any differences in growth rates. Quantifying CFU shows nearly identical growth of wt and Δ*ppe64* in FeCi, whereas the growth of Δ*ppe64* is reduced 85% compared to wt in the presence of Hm (Fig. 1F; Fig. S2). Altogether, these observations demonstrate that the PPE64 channel protein plays a critical role in Hm utilization by the non-albumin pathway.

### The PPE64 channel protein is required for Hm uptake in *Mtb*

Since the PPE64 channel protein is required for growth in Hm, we hypothesized that PPE64 may affect intracellular Hm levels in *Mtb*. For example, a lack of Hm uptake in Δ*ppe64* would result in the absence of iron nutrients for the cell, or the accumulation of intracellular Hm in Δ*ppe64* could result in Hm toxicity, where either mechanism could lead to the absence of growth in Hm as observed in Δ*ppe64*. First, we directly quantified intracellular Hm levels using the pyridine hemochromogen assay (45). Conceptually, harvested cells are first lysed to release Hm, which is reduced to its ferrous iron state and then bound by pyridine. The specific absorbance of the reduced-Hm-pyridine complex at 557 nm can then be monitored to determine Hm levels. Using this assay, we determined that deleting *ppe64* significantly lowers intracellular Hm levels in *Mtb* and that this could be fully restored to wt levels by complementation (Fig. 2A). As an additional control, we also determined Hm levels in strains when grown in the presence of albumin and Hm. Interestingly, albumin significantly stimulates Hm uptake in *Mtb* as Hm levels in wt were ~2-fold higher when compared to the non-albumin condition (Fig. 2B). Since Δ*ppe64* grows in Hm in the presence of albumin (Fig. 1C), expectedly, there was no significant difference in Hm levels between wt and Δ*ppe64*. These results are consistent with the observations that PPE64 is critical for Hm utilization by the non-albumin pathway but has no role in the albumin pathway.

The reduced intracellular Hm levels in Δ*ppe64* suggested that PPE64 may influence Hm uptake in *Mtb*. To test this hypothesis, we examined the susceptibility of Δ*ppe64* to the toxic Hm analog GaPIX. We have previously (30) shown that GaPIX uptake is mediated by Hm uptake components because of their structural similarity. Thus, we reasoned that if Δ*ppe64* has reduced Hm uptake, then it would be less susceptible to GaPIX toxicity due to reduced GaPIX uptake. Conversely, if intracellular Hm is accumulating in Δ*ppe64*, then it would be more susceptible to GaPIX toxicity due to accumulating GaPIX. We observed that the deletion of *ppe64* increases *Mtb* resistance to GaPIX, and susceptibility is fully restored upon complementation (Fig. 2B), which suggests that *ppe64* deletion reduces GaPIX uptake. We also determined the growth pattern of Δ*ppe64* in the presence of increasing levels of Hm. Since excess Hm can be toxic to cells, we reasoned that if Δ*ppe64* accumulates Hm, then Δ*ppe64* growth would not recover in the presence of higher levels of Hm due to increasing Hm accumulation and toxicity. Increasing the Hm concentration to 20 µM and 40 µM has no effect and does not recover Δ*ppe64* growth (Fig. 2C). However, Δ*ppe64* growth recovers nearly 50% at 80 µM Hm and to wt levels at 160 µM Hm (Fig. 2C), suggesting that Δ*ppe64* is not experiencing Hm toxicity.

Finally, we monitored intracellular Hm levels using the HS1-M7A cytoplasmic Hm biosensor, which we previously used in our study to monitor Hm uptake (41). This biosensor has the Hm binding domain of cytochrome *b*_562_ conjugated to a green fluorescent protein (56). During regular Hm uptake, Hm is transported into the cell, and Hm binding by *b*_562_ quenches green fluorescence (GF), whereas during defective Hm uptake, Hm transport into the cell is reduced or absent, and GF is not quenched. The Hm biosensor expression vector pYUB1874 was transformed into wt and Δ*ppe64* strains, and GF was monitored temporally at 24-h intervals. We observed that the GF steadily and significantly increases in Δ*ppe64* over time, indicating that Hm levels are lower in Δ*ppe64* compared to wt (Fig 2D). GF in Δ*ppe64* peaked at ~3-fold higher levels compared to that in wt by day 5, which was visually validated through microscopy analysis (Fig. 2E). Collectively, all of our observations present a convincing case that PPE64 plays a critical role in Hm uptake by the non-albumin Hm utilization pathway in *Mtb*.

### Absence of PPE64 triggers pleiotropic effects on *Mtb* physiology

**Fig 3:**
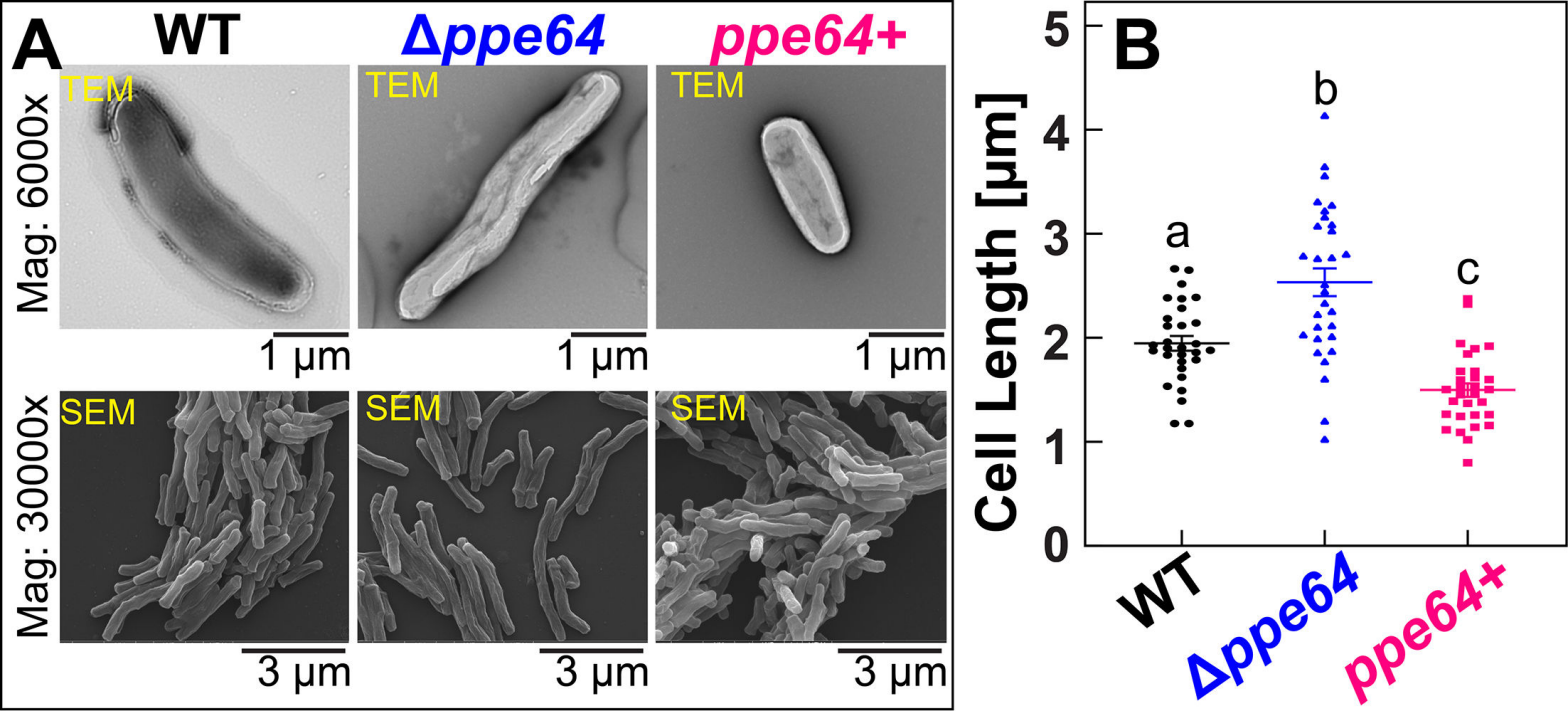
PPE64 influences *Mtb* cellular morphology. (**A** ) Analysis of wt, Δ*ppe64,* and complement strains by TEM and SEM. Strains were grown in standard liquid 7H9 supplemented with 1% glycerol, 10% ADS, and 0.02% tyloxapol. Full uncropped images are provided in Fig. S3. (**B**) Quantification of cell size of wt, Δ*ppe64,* and complement strains from electron microscopy. Three different SEM images for each strain were analyzed in ImageJ, and at least 30 cells for each strain were analyzed to determine cell size. Plots with different lowercase letters indicate statistically significant differences from each other as determined by Tukey’s HSD following an F-test (*P* < 0.002). The source data file is provided.

**Fig 4:**
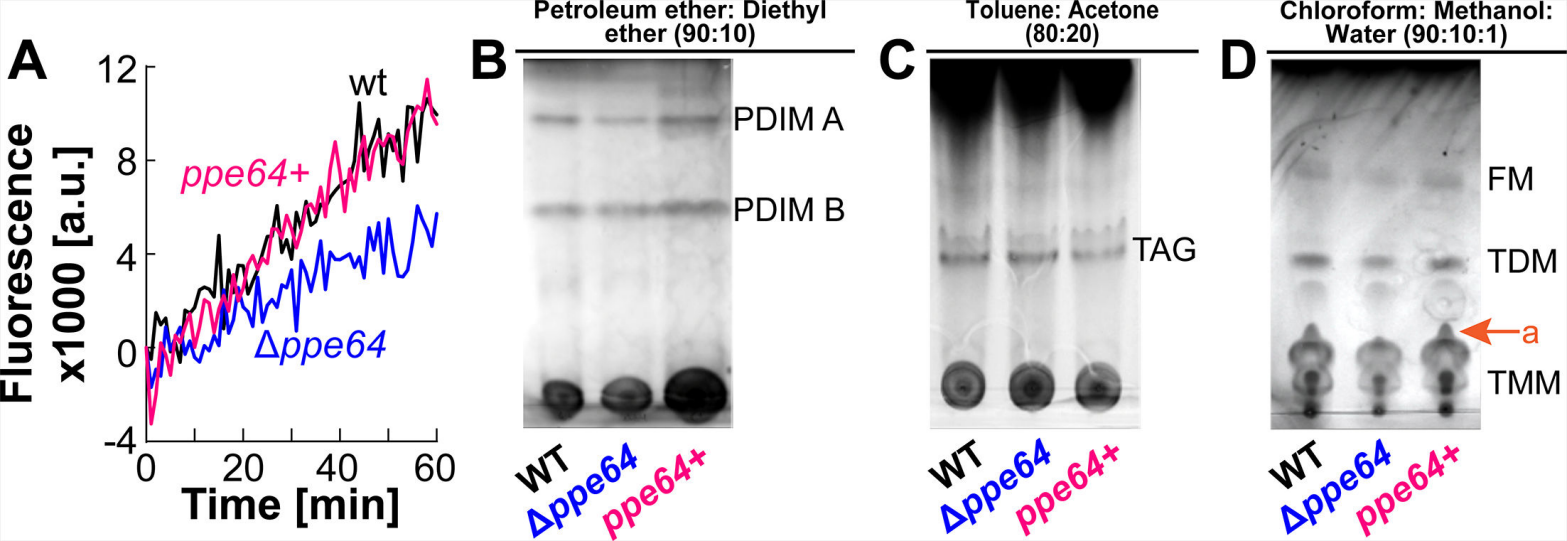
PPE64 influences *Mtb* cell membrane permeability. (**A**) Cell permeability in wt (black), Δ*ppe64* (blue), and complement (pink) strains was monitored by assessing EtBr accumulation. The source data file is provided (**B–D**). Analysis of lipids in *Mtb* strains by TLC. (**B**) Total apolar lipid extracts were resolved by TLC in petroleum ether-diethyl ether (90:10 vol/vol). (**C**) Surface lipids were resolved by TLC in toluene-acetone (80:20 vol/vol) to visualize TAG. TLC experiments were performed with two biological replicates. (**D**) Surface lipids were resolved by TLC in chloroform-methanol-water (90:10:1 vol/vol/vol) to visualize TMM, TDM, and FM. “a” denotes an unknown lipid. Results of biological replicates are shown in Fig. S4. (PDIM: pthiocerol dimycocerosates, FM: free mycolic acids, TDM: trehalose dimycolates, TMM: trehalose monomycolates, TAG: triacylglycerol).

During our microscopic analysis of the Hm biosensor strains, we observed that the Δ*ppe64* cells appeared elongated compared to the wt cells. To further validate these observations, we cultured *Mtb* strains in standard 7H9 liquid medium supplemented with glycerol and ADS and analyzed them by both TEM and SEM (Fig. 3A). WT displayed a cell length range of 1.2–2.5 µm with an average length of 1.9 µm (Fig. 3B). However, deleting *ppe64* increases *Mtb* cell length range to 1.2–4.2 µm with an average length of 2.7 µm with an overall rougher cellular morphology. Interestingly, overproduction of PPE64 in the complement strain significantly reduces the average cell length to 1.5 µm and results in smoother cellular morphology. Since PPE64 is a mycomembrane channel protein that also affects *Mtb* cell shape, we examined whether Δ*ppe64* has altered cell permeability, as this can affect the trafficking of hydrophobic molecules such as Hm. Monitoring membrane permeability by measuring EtBr accumulation (57) showed that deleting *ppe64* reduces the rate of EtBr accumulation, suggesting that Δ*ppe64* has reduced cell membrane permeability (Fig. 4A).

As it is well-established that mycomembrane lipids play a major role in membrane permeability (48, 58), we next examined the cell surface lipid profile of Δ*ppe64*. Strains were cultured similarly, as done for permeability experiments, and apolar and surface lipids were extracted and examined by TLC. Total apolar lipid extracts were resolved in a petroleum ether-diethyl ether solvent system to visualize PDIMs, a major lipid factor that influences *Mtb* membrane permeability. In *Mtb*, during PDIM biosynthesis, two mycocerosates are esterified onto phthiodolone to produce the first PDIM product called PDIM B, which is further reduced and methylated to produce PDIM A (59). Our TLC results from two independent biological experiments show a trend where *ppe64* deletion slightly reduces PDIM A levels (Fig. 4B). Furthermore, we resolved hexane-extracted surface lipids in a toluene-acetone and chloroform-methanol-water solvent systems to visualize triacylglycerol (TAG), FM, and trehalose mono/dimycolates (TMMs, TDMs). While deletion of *ppe64* does not affect TAG (Fig. 4C; Fig. S4B) or FM/TMM/TDM levels (Fig. 4D; Fig. S4C), in the chloroform-methanol solvent system, we observed the loss of an unknown lipid (Fig. 4D; Fig. S4C). Production of this surface-extractable lipid (denoted as “a”) was again restored to wt levels upon complementation. Collectively, our data suggest an important role of the PPE64 channel protein in somehow maintaining cell shape and membrane permeability in conjunction with influencing levels of certain mycomembrane cell surface lipids.

### PPE64 moonlights in Hm iron utilization and biofilm formation

**Fig 5:**
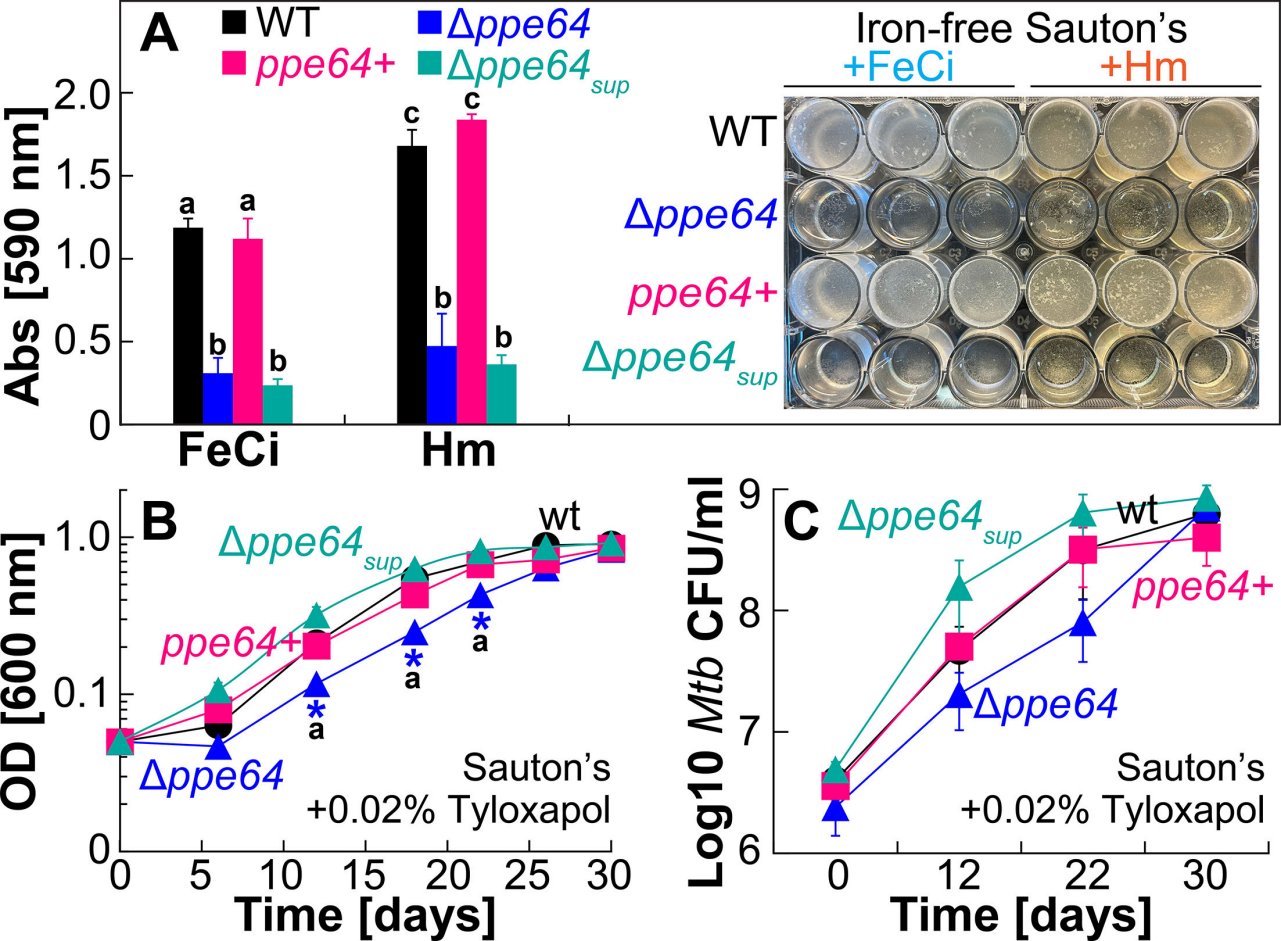
PPE64 influences *Mtb* growth within biofilm independent of the iron source. (**A**) Quantification of biofilm mass in wt (black), Δ*ppe64* (blue), complement (pink), and Δ*ppe64 suppressor* (cyan) strains grown in detergent-free iron-free Sauton’s medium supplemented with either 10 µM FeCi or 10 µM Hm. Biofilms were grown for 5 weeks in 24-well plates and quantified by CV staining. The right panel shows an image of the plate used for biofilm quantification. Plots with different lowercase letters indicate statistically significant differences from each other. (**B**) Growth of wt (black), Δ*ppe64* (blue), complement (pink), and Δ*ppe64 suppressor* (cyan) strains in standard Sauton’s liquid medium (which contains 150 µM FeCi) supplemented with 0.02% tyloxapol. (**C**) Growth of strains in liquid Sauton’s medium at the indicated time points was monitored by determining bacterial CFU counts by plating on 7H10 agar. All error bars represent the SEM of biological triplicates. Asterisks denote that Δ*ppe64* is significantly different from wt at those timepoints. Timepoints with black lowercase letters indicate that there were significant differences between Δ*ppe64* and Δ*ppe64* suppressor strains at those timepoints. Statistical significance was determined by Tukey’s HSD following an F-test (*P* < 0.05). The source data file is provided.

There is compelling evidence that variations in iron (60) and cell surface lipids (10, 61) levels can have significant effects on mycobacterial biofilm formation. Since we observed that deleting *ppe64* affects Hm iron utilization and levels of some mycomembrane lipids, this prompted us to assess whether PPE64 plays any role in *Mtb* biofilm formation. Biofilms were grown for 5 weeks in typical (62) albumin-free detergent-free Sauton’s medium, and biofilm mass was determined by CV staining, but with one specific modification: strains were grown in iron-free Sauton’s containing either 10 µM FeCi or 10 µM Hm to assess the effect of specific iron sources. From the biofilm mass measurements and direct visual examination, it was apparent that wt *Mtb* forms more robust biofilms when Hm is available as an iron source compared to the non-Hm iron ferric citrate (Fig. 5). Based on our observation that Δ*ppe64* cannot utilize Hm iron (Fig. 1E), we expected Δ*ppe64* would not form biofilms in the presence of Hm as the sole iron source. Surprisingly, Δ*ppe64* fails to form the typical pellicular biofilm either in the presence of Hm iron or ferric citrate iron (Fig. 5A), which was fully restored to wt levels upon complementation. This biofilm defect in Δ*ppe64* was specific to the pellicle formation at the air-liquid interface, as visual examination clearly showed growth at the bottom of the wells (Fig. S4D). To ensure that the absence of biofilm in Δ*ppe64* is not a medium-specific effect, we examined the phenotype of all strains grown planktonically in standard Sauton’s medium, which contains ~150 µM ferric citrate, supplemented with tyloxapol. Deleting *ppe64* causes a slight delay in growth in Sauton’s medium (Fig. 5B) similar to what is observed in albumin-free 7H9 medium containing ferric citrate (Fig. 1D). In these growth experiments, statistically significant differences between wt and Δ*ppe64* were observed on days 12, 18, and 22 (Fig. 5B). However, when CFU was enumerated from these timepoints, we did not observe any statistically significant differences (Fig. 5C). These observations demonstrate that while Δ*ppe64* exhibits a slight growth delay under planktonic growth in Sauton’s, it exhibits a significant impairment in forming biofilms when grown statically in Sauton’s medium.

**Fig 6:**
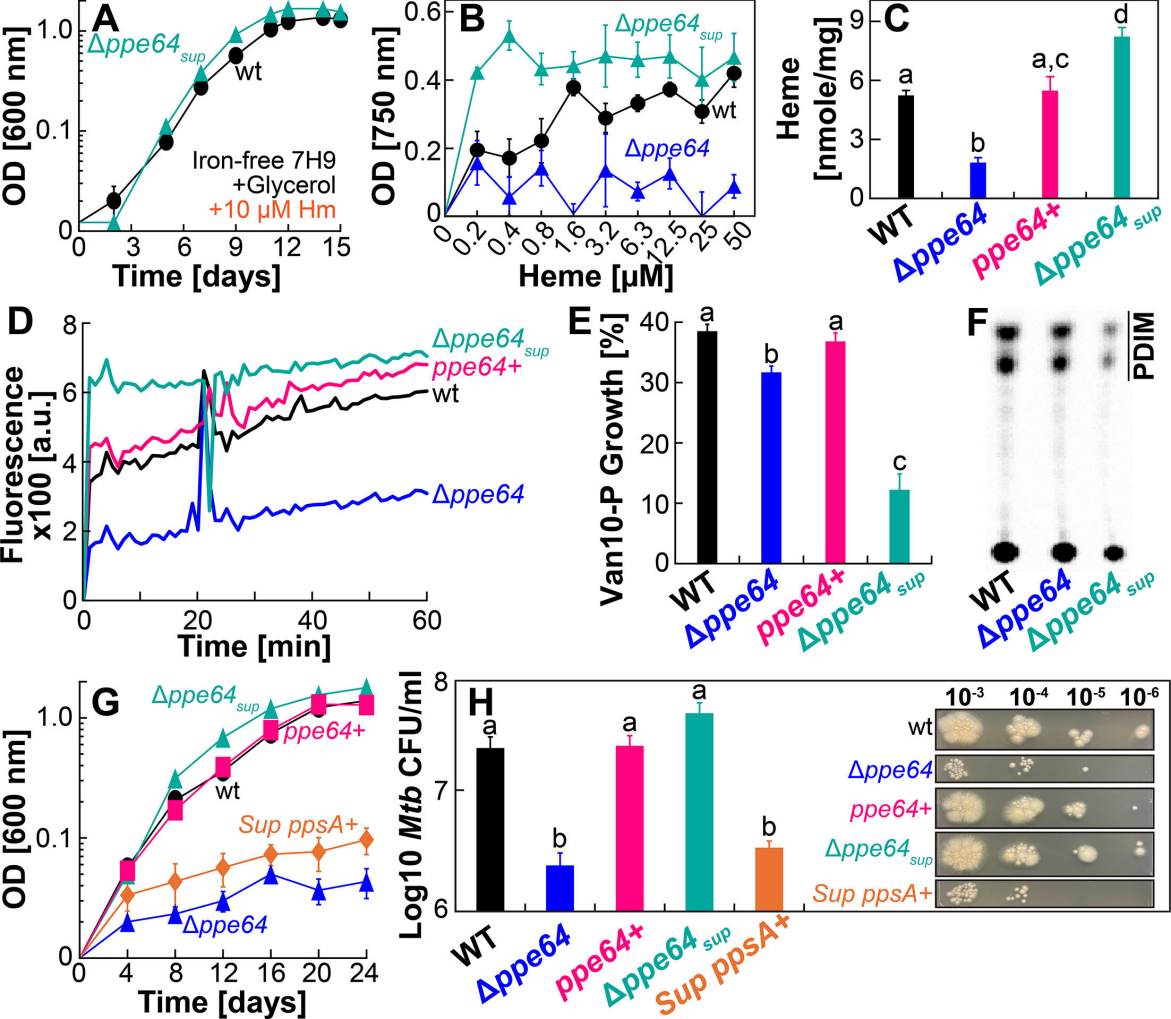
A suppressor mutation restores the ability of the *ppe64* mutant to utilize Hm. (**A**) Growth of wt (black) and Δ*ppe64*-suppressor mutant (cyan) strains in iron-free liquid 7H9 medium supplemented with 10 µM Hm. (**B**) Growth of wt (black), Δ*ppe64* (blue), and Δ*ppe64*-suppressor (cyan) strains in iron-free liquid 7H9 medium containing varying concentrations of Hm. Strains were grown in 96-well plates, and growth was determined by measuring endpoint OD750 on day 20. Full uncropped plate images are shown in Fig. S5A. (**C**) Quantification of Hm levels in strains by hemochromogen assay. (**D**) Cell permeability in strains was monitored by assessing EtBr accumulation. (**E**) Survival of strains in standard liquid 7H9 medium supplemented with 1% glycerol, 10% ADS, and 0.02% tyloxapol in the presence of 10 µg/mL vancomycin determined by the microplate Alamar Blue assay. The growth percent of strains in vancomycin was determined relative to the growth of strains in the absence of vancomycin. (**F**) Analysis of PDIM by TLC in strains labeled with ^14^C-propionate demonstrating the presence or absence of PDIMs. (**G**) Growth of strains in iron-free liquid 7H9 medium containing 1% glycerol, 0.02% tyloxapol, and 10 µM Hm. (**H**) Growth of strains on self-made iron-free solid 7H10 agar plates supplemented with 1% glycerol, 10% ADS, 0.02%, and 10 µM Hm (*n* = 5). The right panel shows a representative image of plates used to determine CFU counts. Uncropped plate images are shown in Fig. S5D. All error bars represent the SEM of a minimum of biological triplicates. For **C**, **E**, and **H**, plots with different lowercase letters indicate significant differences (*P* < 0.04). Statistical significance was determined by Tukey’s HSD following an F-test (*P* < 0.05). The source data file is provided.

In our experiments, while characterizing the growth of Δ*ppe64* in Hm, we made a serendipitous observation, where after ~70 days, Δ*ppe64* abruptly started growing in the non-albumin 7H9 medium containing Hm. This suggested that Δ*ppe64* may have accumulated a suppressor mutation allowing it to start utilizing Hm again in the non-albumin condition. We isolated a single suppressor strain (denoted Δ*ppe64*_sup_) and in growth experiments, verified that this strain is fully capable of utilizing Hm under non-albumin conditions (Fig. 6A) and generally outperforms wt when grown in different concentrations of Hm (Fig. 6B; Fig. S5A). In hemochromogen assays, we validated that Δ*ppe64*_sup_ has significantly high levels of intracellular Hm compared to both wt and Δ*ppe64* (Fig. 6C), which is consistent with its recovered Hm growth phenotype. Identification of this Δ*ppe64*_sup_ Hm suppressor mutant presented a unique opportunity to further examine any connections between the Hm iron utilization and biofilm formation roles of PPE64. We similarly characterized Δ*ppe64*_sup_ in biofilm assays and observed that the recovered Hm-utilizing phenotype in the suppressor does not recover the ability of Δ*ppe64* to form biofilms (Fig. 5A). Moreover, Δ*ppe64*_sup_ also outperforms all other strains when grown planktonically in Sauton’s medium (Fig. 5B and C). These observations convincingly demonstrate that the functions of PPE64 in Hm iron utilization and biofilm formation are discrete and unlinked.

### Reducing PDIM levels increases Hm uptake

To establish a possible mechanism for how Δ*ppe64* could utilize Hm again, we performed further phenotypic characterization of the suppressor strain. As Δ*ppe64*_sup_ has increased intracellular Hm, we first examined its cell membrane permeability by monitoring EtBr accumulation. The EtBr accumulation rate in Δ*ppe64*_sup_ was significantly higher compared to wt, Δ*ppe64,* and complement strains, suggesting that Δ*ppe64*_sup_ has increased membrane permeability (Fig. 6D). As mycomembrane PDIM levels can affect EtBr accumulation rates, we determined PDIM levels in the suppressor strain using two orthogonal methods. First, we performed a Van10-P vancomycin susceptibility assay, which was recently (50) established as a straightforward and highly effective method to assess PDIM levels. In this assay, increased susceptibility to vancomycin strongly correlates with reduced PDIM levels. The Van10-P assay showed that Δ*ppe64*_sup_ is far more susceptible to vancomycin compared to all other strains, suggesting that the suppressor has reduced PDIM levels (Fig. 6E). Second, we confirmed the Van10-P observations by directly assessing PDIM levels, which demonstrated that PDIM levels are indeed reduced in the suppressor strain (Fig. 6F). These observations suggested that the suppressor strain may have accumulated mutations that result in reduced PDIMs. As such, using whole-genome sequencing, we identified two non-synonymous (*rv1611* and *rv2374c*), one synonymous (*rv3152c*), and one frameshift mutation (*rv2931*) (Fig. S5B and C) in the suppressor mutant strain. Of these four mutations, the most consequential mutation is the frameshift mutation in *rv2931*, which encodes PpsA, a multifunctional protein that plays a key role in initiating PDIM biosynthesis (63, 64). In Δ*ppe64*_sup_, a single-nucleotide insertion in *ppsA* results in an opal mutation (premature TGA stop codon, Fig. S5C), suggesting that the production of a truncated PpsA may lead to reduced PDIM levels as observed in Δ*ppe64*_sup_. We hypothesized that reduced PDIM levels in Δ*ppe64*_sup_ may allow cells to again traffic Hm, alleviating the Hm utilization defect of Δ*ppe64*. If this was the case, then reproducing PDIMs in Δ*ppe64*_sup_ should again prevent the cells from utilizing Hm. To test this hypothesis, we overexpressed wt *ppsA* in Δ*ppe64*_sup_ using the episomal vector pOAL410. First, we confirmed that PDIM is reproduced in Δ*ppe64*_sup_ by assessing PDIM levels (Fig. S5D) and measuring susceptibility of strains to vancomycin in Van10-P assays (Fig. S5E). Next, we determined the ability of strains to utilize Hm. Expectedly, reproduction of PpsA in the suppressor (Sup *ppsA*+) prevents Δ*ppe64*_sup_ from growing in the presence of Hm (Fig. 6G and H ; Fig. S5F), confirming our hypothesis. Collectively, our observations suggest that reduced PDIM levels in Δ*ppe64*_sup_ increase membrane permeability, which allows Δ*ppe64* to again traffic and utilize Hm.

### PPE64 has two oligomeric states with different properties

**Fig 7:**
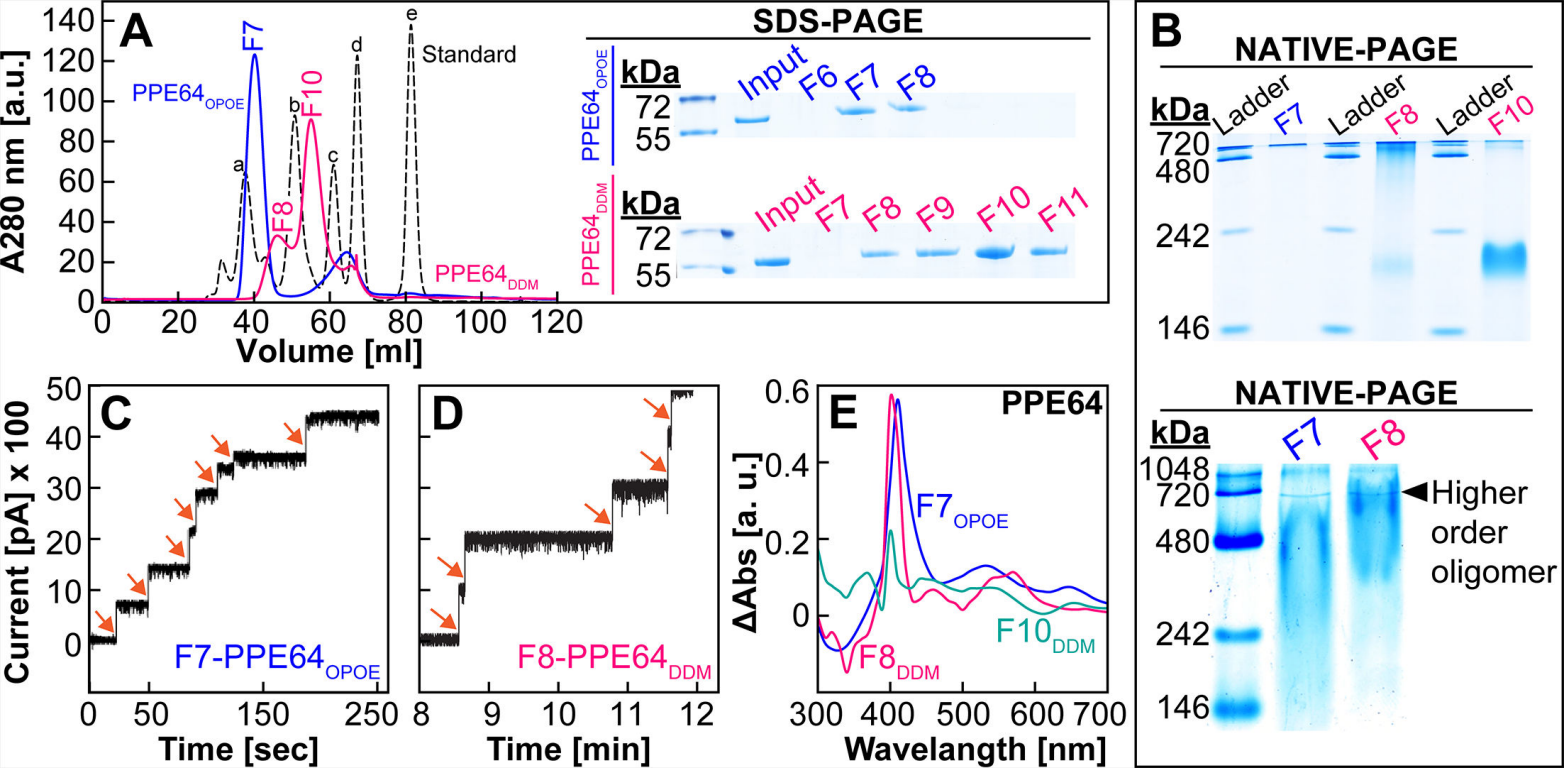
The higher-order oligomeric state of PPE64 forms water-filled membrane channels. (**A** ) Analysis of PPE64 refolded in OPOE (blue) or DDM (pink) by SEC using Sepax SRT-10C SEC 300 column. Colored numbers within the chromatogram show the fraction number. Bio-Rad standard (black dotted line) peaks: a— 670 kDa, b—158 kDa, c—44 kDa, and e—1.3 kDa. The right panel in A shows analysis of SEC fractions by SDS-PAGE. PPE64 monomer is ~64 kDa. (**B**) Analysis of SEC fractions by NATIVE-PAGE showing two oligomeric states of PPE64. F7 (OPOE) and F8 (DDM): 480-720 kDa; F10 (DDM): 146–242 kDa. (**C and D**) Channel-forming activity of PPE64 protein fractions in planar lipid bilayers. Proteins were added to DphPC membranes with 50 mV applied potential, and then the current trace was recorded for PPE64-F7_OPOE_ (**C**), PPE64-F8_DDM_ (**D**), and PPE64-F10_DDM_ (Fig. S6C). Each stepwise increase (orange arrows) in the current trace represents a protein-mediated channel formation in the lipid bilayer. Y-axis scales are the same in (**C–E**). Detection of Hm binding by PPE64 SEC fractions through difference absorption spectroscopy. Free heme spectra were subtracted from heme-incubated protein spectra at protein concentrations of 10 µM. The source data file is provided. For (**C and D**), source data acquisition files (axon binary files) are too large for upload and require pClamp software for viewing and will be provided upon request. Full uncropped images of all protein gels are shown in Fig. S6.

In our previous study (41), we established a detailed process of purifying and refolding the PPE64 mycomembrane protein in the presence of the detergent OPOE (n-Octyl-oligo-oxyethylene). Subsequently, in electrophysiology experiments with planar lipid bilayers with specific controls, we demonstrated that PPE64 forms water-filled channels (41). Our observations showing that PPE64 plays separable roles in Hm iron utilization and biofilm formation suggested that PPE64 may have different protein folded states or domains functioning in these distinct processes, prompting us to further examine the properties of PPE64. We purified recombinant PPE64 and then tested refolding of PPE64 in different detergents such as OPOE (control), DDM (n-dodecyl-β-D-maltoside), and OG (octyl glucoside). While we did not achieve any refolding in OG, PPE64 could be refolded in DDM, similar to OPOE. Analysis of refolded PPE64 by size exclusion chromatography (SEC) showed that PPE64 is refolded into a single oligomeric state in OPOE and in two different oligomeric states in DDM, where all SEC fractions showed the presence of monomeric PPE64 (~62 kDa) as visualized by denaturing PAGE (Fig. 7A; Fig. S6). Analysis of SEC fractions by non-denaturing PAGE confirmed that OPOE refolds PPE64 into a single oligomeric state (fraction F7) of ~480–720 kDa, and DDM refolds PPE64 into two oligomeric states of ~480–720 kDa (F8) and ~146–242 kDa (F10), where the F10 oligomer is the predominant species in DDM (Fig. 7B; Fig. S6). We next examined the channel-forming properties of all PPE64 oligomers in electrophysiology experiments as we have done before (41). We formed a lipid membrane in the aperture of a Delrin cup using DphPC lipids, a specific PPE64 oligomer was added to the *cis* compartment, and the current trace was temporally monitored. F7 of PPE64_OPOE_ rapidly was inserted into the lipid bilayer and formed channels with an average conductance of ~14 nS (Fig. 7C), which is in line with our previous observations (41). F8 of PPE64_DDM_ inserted into the lipid bilayer at a slower rate and formed channels with an average conductance of ~20 nS (Fig. 7D). In contrast, F10 of PPE64_DDM_ did not form any pores (Fig. S6C). We also determined Hm-binding capability of these PPE64 oligomers through absorption spectroscopy by monitoring the presence of the characteristic Soret peak at ~410 nm, indicative of protein-Hm binding. F7 of PPE64_OPOE_ exhibits a strong Soret peak indicative of Hm binding (Fig. 7E), recapitulating our previous observations (41). F8 of PPE64_DDM_ similarly exhibits a strong Soret peak; however, F10 of PPE64_DDM_ has significantly reduced absorbance at 410 nm indicative of very weak interactions with Hm (Fig. 7E). Collectively, these observations demonstrate that the lipidomic environment can direct the formation of at least two oligomeric states in PPE64 and that the higher-order oligomeric state is responsible for channel activity and interacting with Hm.

### PPE64 is important for *Mtb* growth in human macrophages

**Fig 8:**
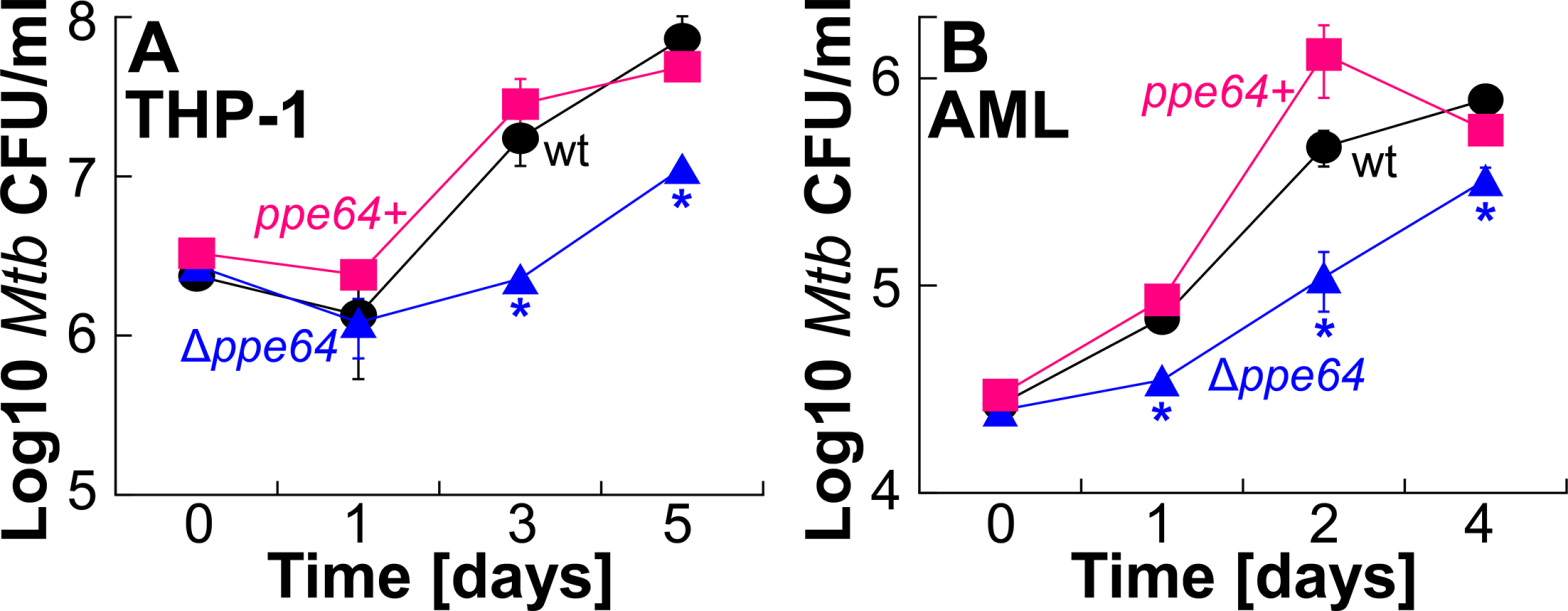
PPE64 influences *Mtb* growth within macrophages. (**A**) THP-1 monocytes were differentiated into macrophages with PMA and then infected with *Mtb* strains at an MOI of 10. Macrophages were lysed at the indicated time points, and *Mtb* viability was determined by enumerating CFU on agar plates. (**B**) AMLs were infected with *Mtb* strains at an MOI of 2. Infected AMLs were lysed at the indicated time points, and *Mtb* viability was determined by enumerating CFU on agar plates. All error bars represent the SEM of biological triplicates. Asterisk denotes that Δ*ppe64* is significantly different from wt at those timepoints. Statistical significance was determined by Tukey’s HSD following an F-test (*P* < 0.05). Source data file is provided.

Our observations show that PPE64 affects multiple aspects of *Mtb* physiology: Hm iron utilization, biofilm formation, cell shape, and membrane permeability. As such, we wanted to determine whether PPE64 contributes to virulence by examining the growth of *Mtb* within macrophages. For macrophage infection experiments, we constructed a separate Δ*ppe64* complement strain by using the integrative expression vector pDM106 (Table S2), which expresses *ppe64* using its native promoter. In infection experiments with THP-1 cells differentiated into macrophages, deletion of *ppe64* significantly reduced *Mtb* survival at 3 and 5 days post-infection, with a trend showing slow recovery in Δ*ppe64* (Fig. 8A). We also examined the growth of *Mtb* in AMLs generated from human PBMCs. A recent study by the Schlesinger (53) group demonstrated that AMLs derived from human PBMCs serve as an excellent simplified *in vivo* proxy for studying *Mtb* infections. We generated high-purity AMLs from human PBMCs and validated purity using flow cytometry by CD11c and HLA-DR staining. We routinely achieved 95%–99% AML purity, as shown in the representative flow cytometry plot (Fig. S6D). A noticeable difference was that AMLs are more permissive to *Mtb* growth relative to differentiated THP-1 cells. Following 24 h post-infection, while wt *Mtb* exhibits an initial reduction in growth in THP-1, it proliferates within AMLs (Fig. 8B). This pattern was apparent for all *Mtb* strains in both infection conditions. Deleting *ppe64* also significantly reduced growth of *Mtb* in AMLs with a similar trend of recovery (Fig. 8B). The *ex vivo* growth defect of Δ*ppe64* recovered to near wt levels upon complementation. Collectively, these observations demonstrate that under these experimental conditions, PPE64 plays an important role in *Mtb* survival within human macrophages.

## DISCUSSION

Our study started with the goal of characterizing the role of the PPE64 mycomembrane channel protein in Hm iron utilization. We know that *Mtb* has at least two means of acquiring Hm iron, termed the non-albumin- and albumin-dependent pathways (29). Under the experimental conditions examined in our study, we discovered that PPE64 is a major requirement for *Mtb* Hm utilization by the non-albumin pathway at Hm concentrations of 10, 20, and 40 µM (Fig. 1 and 2C) and that it is important for Hm uptake (Fig. 2). The requirement for PPE64 also appears to be conditional, because the *ppe64* mutant grows when Hm level is increased to 80 and 160 µM (Fig. 2C). Since Hm is highly reactive, free Hm is extremely rare in the human body and is sequestered within hemoproteins. Thus, it is debatable whether *Mtb* would ever encounter free Hm levels of 80–160 µM under physiologically relevant conditions in the host. But it is conceivable that *Mtb* may perhaps use a low-affinity Hm transporter(s) at high Hm levels (80–160 µM), whereas PPE64 functions in Hm transport at lower levels of Hm. Alternatively, 80–160 µM Hm may be high enough that Hm overcomes the PDIM barrier in the mycomembrane, bypassing the need for PPE64, allowing Δ*ppe64* to grow. Regardless, our data clearly establish an important role of PPE64 in trafficking Hm into the *Mtb* cell by the non-albumin pathway.

Through our microscopy analysis, we observed that PPE64 also influences *Mtb* cellular morphology, where in its absence, cells are more elongated, and PPE64 overproduction leads to smaller cell size (Fig. 3). To the best of our knowledge, this is the first evidence of a PPE protein affecting cell size. It is known that during cell division, mycobacterial cells elongate asymmetrically at the poles, producing daughter cells of different size, growth rate, and cell wall composition (65–68). There is ample evidence demonstrating that the spatiotemporal organization of OMPs and interactions with peptidoglycan play important roles in cell division in diderm bacteria (69–73). Moreover, the outer membrane channel protein OmpA (74) in gram-negative bacteria plays a critical role in interacting with other OMPs and LPS to order and stabilize the outer membrane (70, 75). These observations raise important questions about whether the *Mtb* PPE64 channel protein could have functions in influencing mycomembrane stability or the mycobacterial cell division apparatus and opens exciting new avenues of research for future studies. We also observed that the absence of PPE64 renders *Mtb* cells less permeable to EtBr (Fig. 4A), which can sometimes be a consequence of altered lipid levels in the mycomembrane. In our lipidomic analyses, while we observed a trend of slight reduction of PDIM A levels in the *ppe64* mutant (Fig. 4B), it must be noted that these differences could be a result of variations in sample loading as observed between the two experimental replicates (Fig. S4A). The trend of reduced PDIM levels is consistent with slightly increased susceptibility to vancomycin as observed in Δ*ppe64* (Fig. 6E). This is in line with recent findings showing that the loss of PDIM increases susceptibility to vancomycin (50). However, the trend of slightly reduced PDIM A in Δ*ppe64* is not due to the random loss of PDIM production that can happen in *Mtb* cultures *in vitro* (76, 77), as complementation reproduces PDIM A to wt levels. Furthermore, loss of PDIM increases cell permeability (58) and EtBr accumulation (50), which is not the case in Δ*ppe64*, suggesting that the marginal change in PDIM A level is not the cause of reduced EtBr accumulation in Δ*ppe64*. In a recent study, Rodrigues et al. demonstrated that loss of the mycomembrane porin MspA reduces EtBr accumulation in *M. smegmatis* (78). Since PPE64 is a mycomembrane channel protein in *Mtb* (41) (Fig. 7C and D), it is conceivable that PPE64 could traffic EtBr across the mycomembrane and hence its absence reduces EtBr accumulation in Δ*ppe64*. Altogether, our data further establish a link between mycobacterial PPE proteins, PDIM levels, and membrane permeability, which has been shown in previous studies (34, 79). Nonetheless, we made a serendipitous discovery that PDIM levels can influence Hm uptake because the Δ*ppe64* suppressor mutant, which can utilize Hm, produces far less PDIMs compared to wt and Δ*ppe64* (Fig. 6F). Reproducing PDIM levels (Fig. S5D and E) in the Δ*ppe64* suppressor mutant recapitulated the Hm utilization defect (Fig. 6G and H), demonstrating that PDIM levels in the mycomembrane can influence Hm uptake and utilization. It should be noted that we did not undertake a second attempt to isolate a suppressor mutant, and thus we do not know whether reducing PDIMs is a general response to alleviate the loss of Hm utilization phenotype in Δ*ppe64*. In our TLC analysis, we also observed that an unknown surface lipid (Fig. 4D, denoted “a”) was absent in Δ*ppe64,* and we do not know whether this lipid could influence membrane permeability. A limitation of our lipidomic studies is that analysis was conducted only on strains grown in the typical standard 7H9 growth medium containing albumin. Since PPE64 is essential for Hm utilization in the absence of albumin, a key future study will be to comprehensively characterize the mycomembrane lipid composition of *Mtb* strains in medium with and without albumin and in different iron sources.

The most consequential finding of our study is that PPE64 has discrete moonlighting functions in Hm iron acquisition and biofilm formation in *Mtb*. Under planktonic growth conditions, PPE64 is essential for Hm iron utilization by the non-albumin pathway (Fig. 1E). Whereas, under biofilm (static) growth conditions, PPE64 is important for the typical *Mtb* pellicular biofilm formation (Fig. 5A). The role of PPE64 in biofilm formation is independent of the iron source as the Δ*ppe64* suppressor mutant, which can utilize Hm, remains defective in biofilm formation. This bifunctional nature of the PPE64 channel protein is reminiscent of bifunctional outer membrane channel proteins of *Shigella* and *E. coli* that function in discrete processes such as motility, adhesion, and biofilm formation depending on the host niche. For example, the dual-functioning outer membrane channel protein IcsA is crucial for actin-based motility (80) and biofilm formation (81) depending on the stage of the *S. flexneri* infection cycle. In *E. coli*, the outer membrane channel proteins AIDA (82) and Ag43 (83) have diverse functions in cell aggregation, cell-to-cell contact, and biofilm formation. Another prime example is the dual functioning outer membrane channel protein OmpM of gram-negative Firmicutes, which is crucial for nutrient uptake and tethering the outer membrane to the cell wall (84). Thus, there is ample precedence to suggest that the distinct functions of PPE64 may also be host niche-specific. For example, macrophages play a key role in Hm recycling (85), and a significant part of the *Mtb* life cycle is within macrophages. As such, *Mtb* could be using PPE64 to acquire host Hm iron during intracellular growth. While our observations from infected macrophages (Fig. 8) seem to support this hypothesis, we cannot irrefutably state that the impairment is solely due to loss of Hm acquisition or from other pleiotropic effects in Δ*ppe64*. Of importance, we observed that AMLs are more permissive to *Mtb* growth than THP-1 cells, in line with previous findings (53, 86–89). Since THP-1 monocytes serve as precursors for many subsets of macrophages, this could explain the constrained growth of *Mtb* in THP-1 macrophages. A limitation of these macrophage experiments is that we can only assess how PPE64 affects intracellular *Mtb* growth. Ample evidence demonstrates that extracellular *Mtb* (i) form biofilms in mice, non-human primates, and human lungs, leading to drug tolerance (8), (ii) form biofilms on airway epithelial cells (6), and (iii) clinical *Mtb* isolates upregulate *ppe* family genes during biofilm growth (90). Thus, PPE64 may also be an important factor for *Mtb* biofilm-dependent growth in the host during its extracellular life cycle.

Dissecting the bifunctional roles of PPE64 and their specific contributions to *Mtb* survival within the host is not a trivial matter, as this requires carefully controlled experiments, use of appropriate infection models, and most importantly, structural knowledge of PPE64. Our biochemical characterization clearly shows that PPE64 (~62 kDa monomer) has at least two oligomeric states (Fig. 7). The higher-order oligomer (~480–720 kDa, predicted nonamer) forms channels in membranes and exhibits strong Hm binding, and thus we hypothesize that this oligomeric state functions in Hm utilization (Fig. 1E) and uptake (Fig. 2). The lower-order oligomer (~146–242 kDa, predicted trimer) does not form channels or bind Hm and may be the oligomeric state that functions in biofilm-dependent growth. We fully recognize that these hypotheses must be validated with detailed structural examination of PPE64 and that the findings of our study only begin to address the many unanswered questions surrounding how mycobacterial PPE proteins function in *Mtb* physiology. Most importantly, our previous (41) and current studies present conclusive evidence that PPE64 is a novel mycomembrane channel protein that plays major roles in mycobacterial physiology and contributes to *Mtb* growth *ex vivo*. The localization of PPE64 in the cell surface, its contribution to *Mtb* virulence, and its presence only in pathogenic mycobacteria make it an appealing candidate for developing highly targeted chemotherapy. In conclusion, we believe our study presents exciting new findings that will open new avenues of research for the field.

## Data Availability

All source data files are provided and/or publicly available and are also available to anyone upon request. Whole-genome sequencing data files are available through the NCBI Sequence Read Archive (BioProject ID PRJNA1272022). Lipid bilayer data acquisition files (axon binary file) require pClamp software, which, to the best of our knowledge, is only available for purchase through Molecular Devices. All requests should be addressed to Dr. Avishek Mitra.

## SUPPLEMENTAL MATERIAL

Supplemental material mbio.03281-25-s0001.pdf Supplemental figures and tables.

ASM does not own the copyrights to Supplemental Material that may be linked to, or accessed through, an article. The authors have granted ASM a non-exclusive, world-wide license to publish the Supplemental Material files. Please contact the corresponding author directly for reuse.
