# Supplementary material for: PPE64 is a mycomembrane channel protein that functions in heme iron uptake and moonlights in biofilm formation in Mycobacterium tuberculosis
Source: mBio. 2025 Dec 11;17(1):e03281-25. doi: 10.1128/mbio.03281-25 (PMC12802285; doi:10.1128/mbio.03281-25)
Supplement: Supplemental material — Supplemental figures and tables. [file mbio.03281-25-s0001.pdf]

SUPPLEMENTARY INFORMATION

PPE64 is a mycomembrane channel protein that functions in heme iron uptake and moonlights in biofilm formation in *Mycobacterium tuberculosis*

Padam Singh[1], Charles B. Kaufman[2], Lisa Whitworth[3], Reed M. Stubbendieck[4], Randy Morgenstein[5], Karen L. Wozniak[2], and Avishek Mitra[1]*

[1] Department of Microbiology and Molecular Genetics, Oklahoma State University, 318 Life Science East, Stillwater, OK 74078, USA

[2] Department of Microbiology and Molecular Genetics, Oklahoma State University, 405 Life Science East, Stillwater, OK 74078, USA

[3] OSU Microscopy Laboratory, Oklahoma State University, 1110 S. Innovation Way, Stillwater, OK 74078, USA

[4] Department of Microbiology and Molecular Genetics, Oklahoma State University, 314 Life Science East, Stillwater, OK 74078, USA

[5] Department of Microbiology and Molecular Genetics, Oklahoma State University, 418 Life Science East, Stillwater, OK 74078, USA

Running title: PPE64 affects *Mycobacterium tuberculosis* physiology

Keywords: *Mycobacterium tuberculosis*, iron acquisition, heme, biofilm, outer membrane, mycomembrane channel protein, PPE

*Address correspondence to: Avishek Mitra, avi.mitra@okstate.edu

SUPPLEMENTARY FIGURES

Supplementary figure 1.

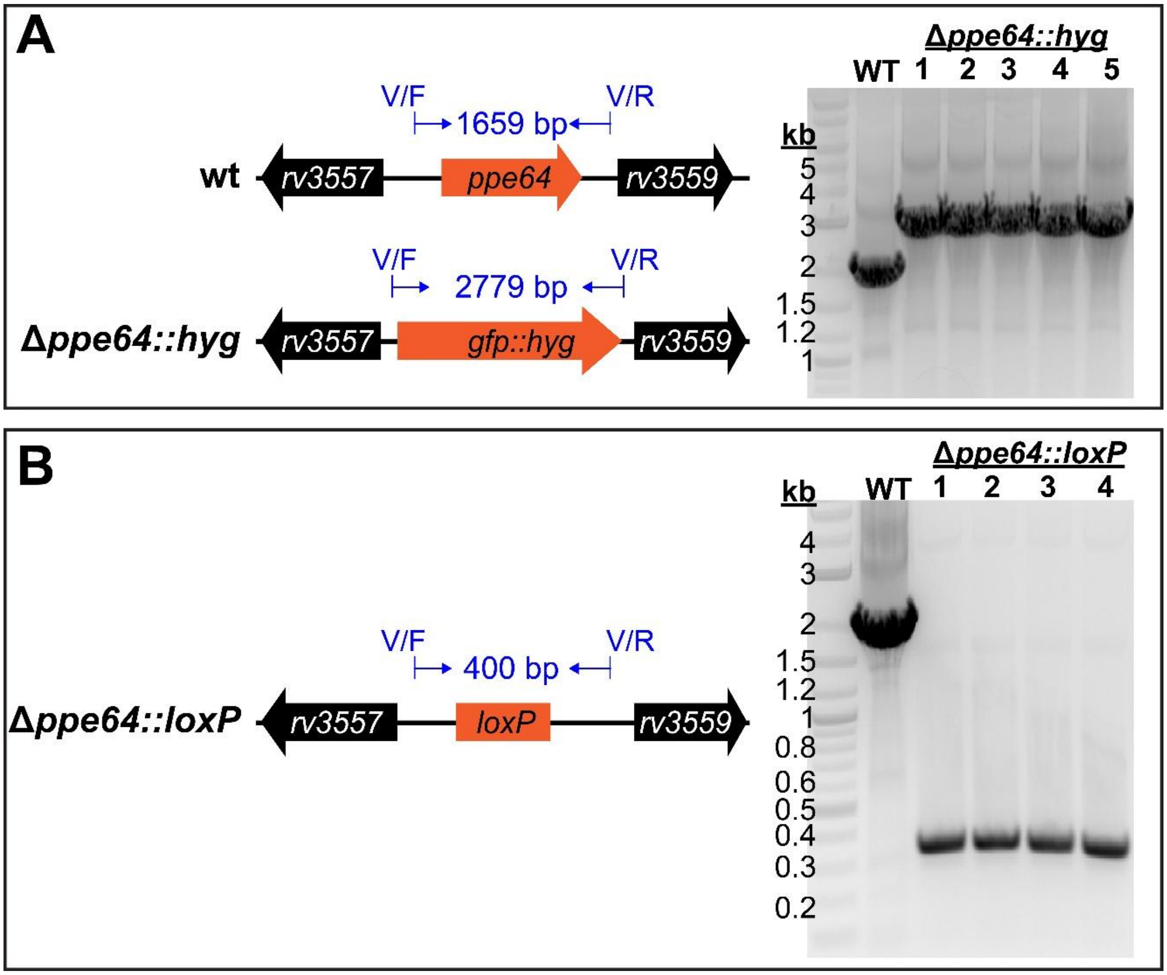

Figure S1. Construction of *ppe64* isogenic deletion strain.
Schematic representation of the *Mtb ppe64* locus and PCRs performed to validate marked (**A**) and unmarked (**B**) deletion mutants of *ppe64*. Colored primer pair shows expected product length in wt, marked and unmarked strains.

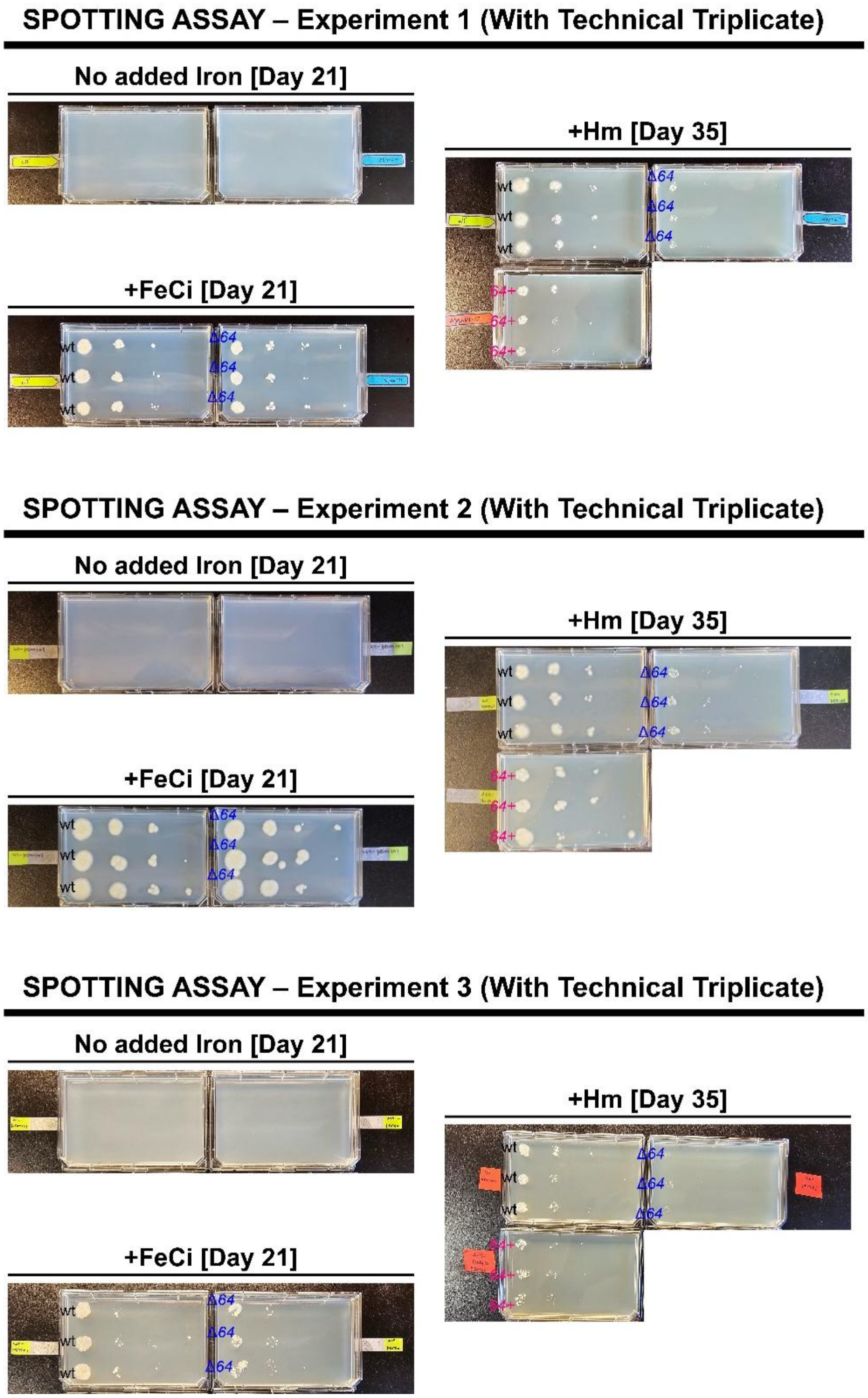

Figure S2. Spotting assay plates used for CFU counts as shown in figure 2.
Growth of wt, Δ*ppe64* and complement (*ppe64*+) strains on self-made iron-free solid 7H10 agar plates which has been supplemented with either no additional iron or 10 μM FeCi or 10 μM Hm. The no added iron and Hm agar plates contain 20 μM of the iron chelator 2'2-dipyridyl (DIP) to prevent utilization of trace iron. Single cell suspension of iron-depleted strains were normalized to OD_{600} 0.05, then serially diluted and 5 μl of each dilution was spotted on agar plates. No added iron and FeCi agar plates were imaged on day 21 and Hm agar plates were imaged on day 35. Spotting assay experiments were performed in biological triplicate and each biological replicate contained technical replicates..

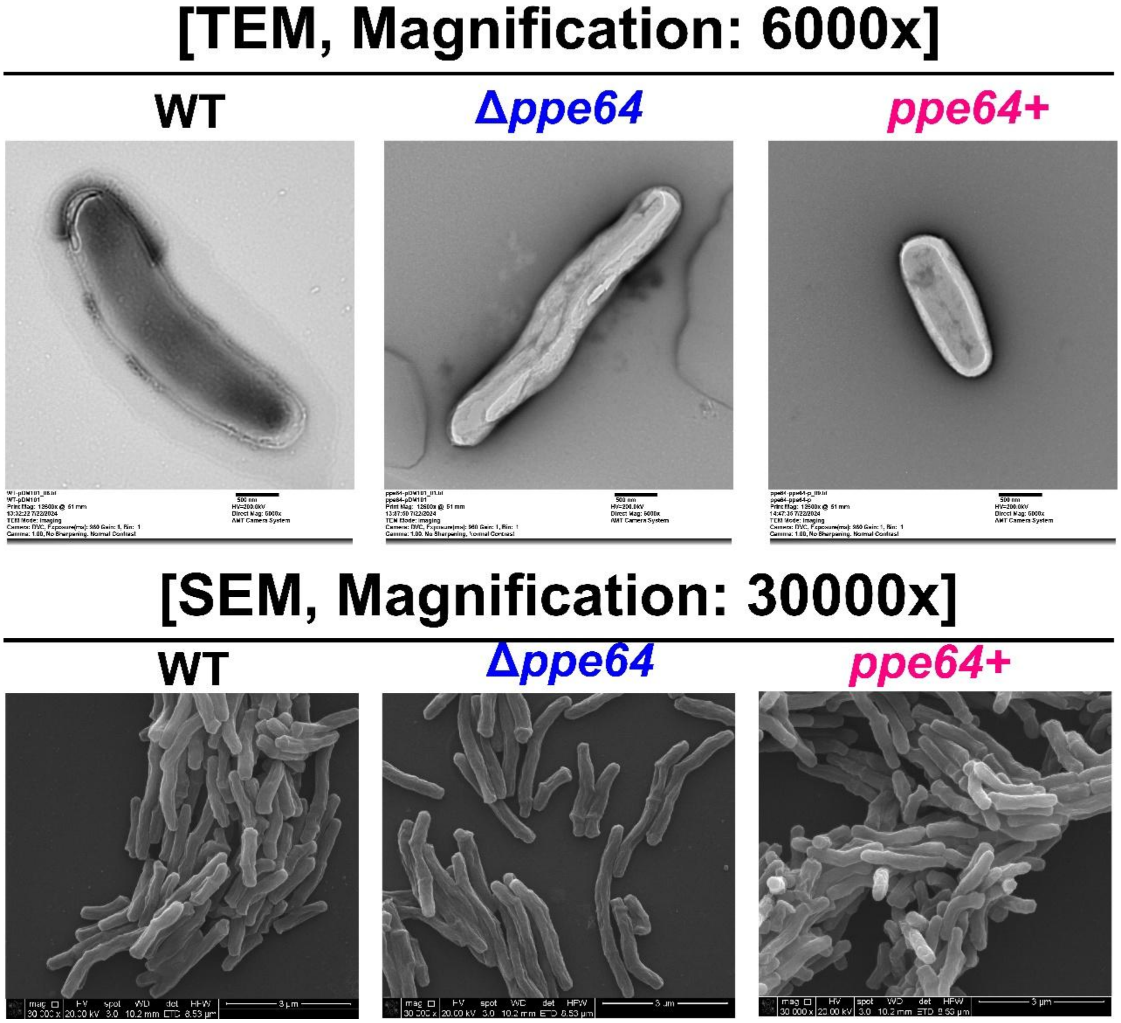

Figure S3. Uncropped TEM & SEM images of strains that are shown in figure 4A.

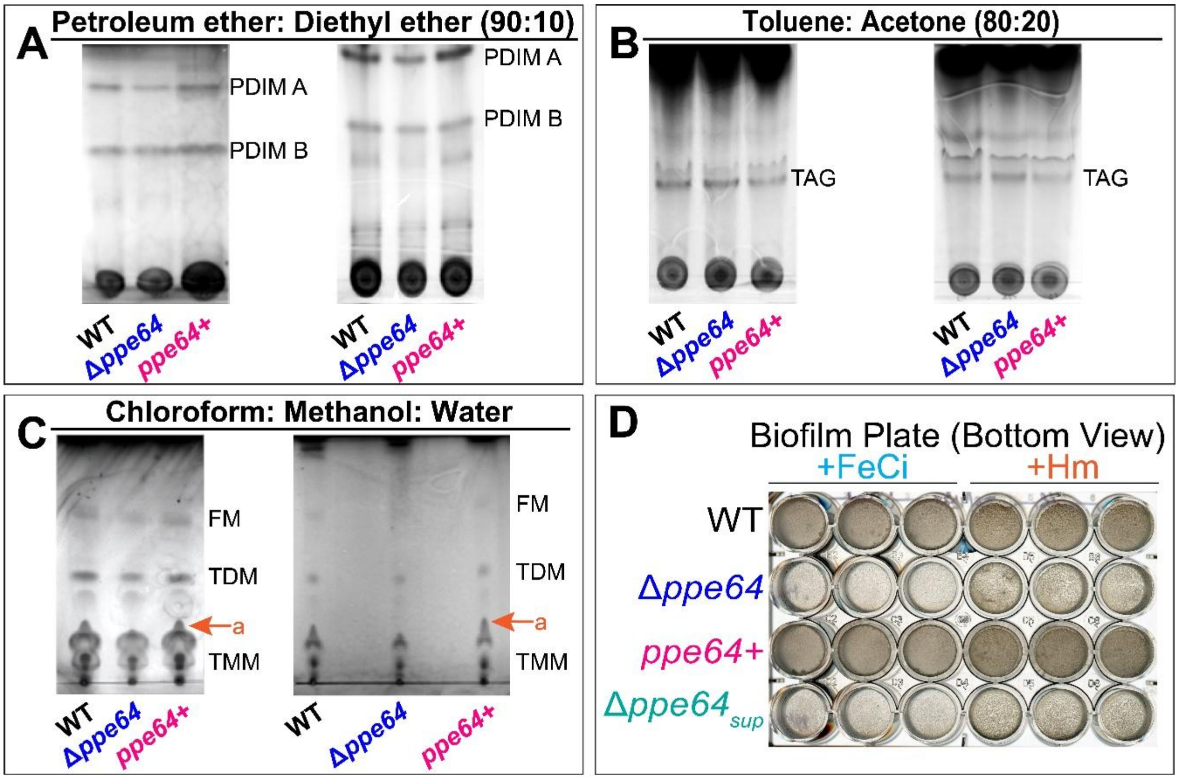

Figure S4. Thin layer chromatography and biofilm formation.

A-C. Biological replicates of TLC that are shown in figure 4. **D.** Bottom view of biofilm plate shown in figure 5A.

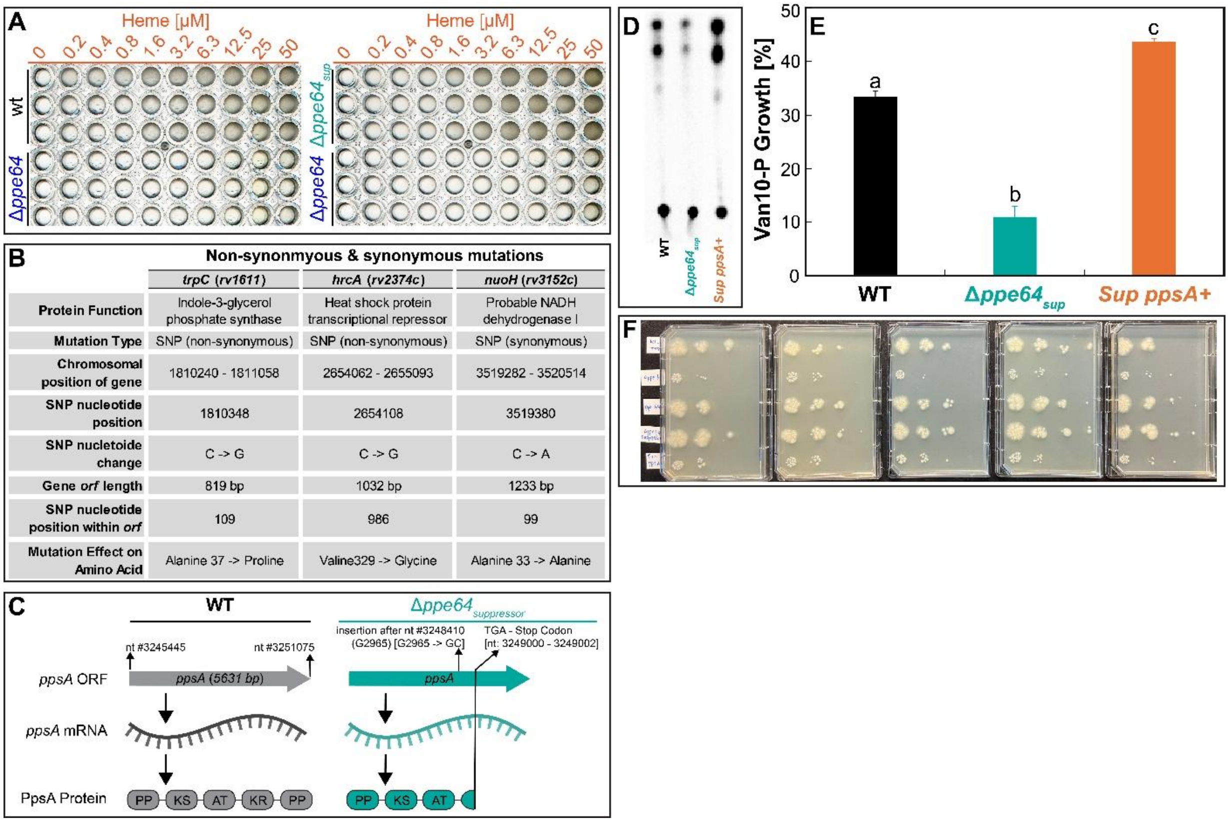

Non-synonmyous & synonymous mutations	*trpC* (*rv1611*)	*hrcA* (*rv2374c*)	*nuoH* (*rv3152c*)
Protein Function	Indole-3-glycerol phosphate synthase	Heat shock protein transcriptional repressor	Probable NADH dehydrogenase I
Mutation Type	SNP (non-synonymous)	SNP (non-synonymous)	SNP (synonymous)
Chromosomal position of gene	1810240 - 1811058	2654062 - 2655093	3519282 - 3520514
SNP nucleotide position	1810348	2654108	3519380
SNP nucleotoide change	C -> G	C -> G	C -> A
Gene *orf* length	819 bp	1032 bp	1233 bp
SNP nucleotide position within *orf*	109	986	99
Mutation Effect on Amino Acid	Alanine 37 -> Proline	Valine329 -> Glycine	Alanine 33 -> Alanine

Figure S5. Characterization of Δ*ppe64* suppressor strain.

A. Uncropped images of 96-well plates used for growth experiment shown in figure 5. **B.** Non-synonymous and synonymous mutations in the Δ*ppe64* suppressor strain. **C.** Frameshift mutation in the Δ*ppe64* suppressor strain. A cytosine insertion after nucleotide 3248410 (Guanine 2965 in *ppsA* open reading frame) causes a frameshift mutation resulting in a pre-mature TGA stop codon. This likely results in production of truncated PpsA protein in the suppressor. PpsA protein domains: **PP** – phosphopantetheinylate acyl carrier protein, **KS** – ketosynthase, **AT** – acyltransferase, **KR** – ketoreductase. **D.** Analysis of PDIM by TLC in strains labelled with ^{14}C-propionate demonstrating presence or absence of PDIMs. **E.** Survival of strains in standard liquid 7H9 medium supplemented with 1% glycerol, 10% ADS and 0.02% tyloxapol in the presence of 10 μg/ml vancomycin determined by the microplate alamar blue assay. Growth percent of strains in vancomycin was determined relative to the growth of strains in the absence of vancomycin. All error bars represent SEM of biological triplicates. Plots with different lowercase letter indicates significant differences ($p<0.001$). Statistical significance was determined by Tukey's HSD following an F-test ($p < 0.05$). Source data file is provided. **F.** Uncropped images of agar plates (n=5) used for growth experiment shown in figure 6H.

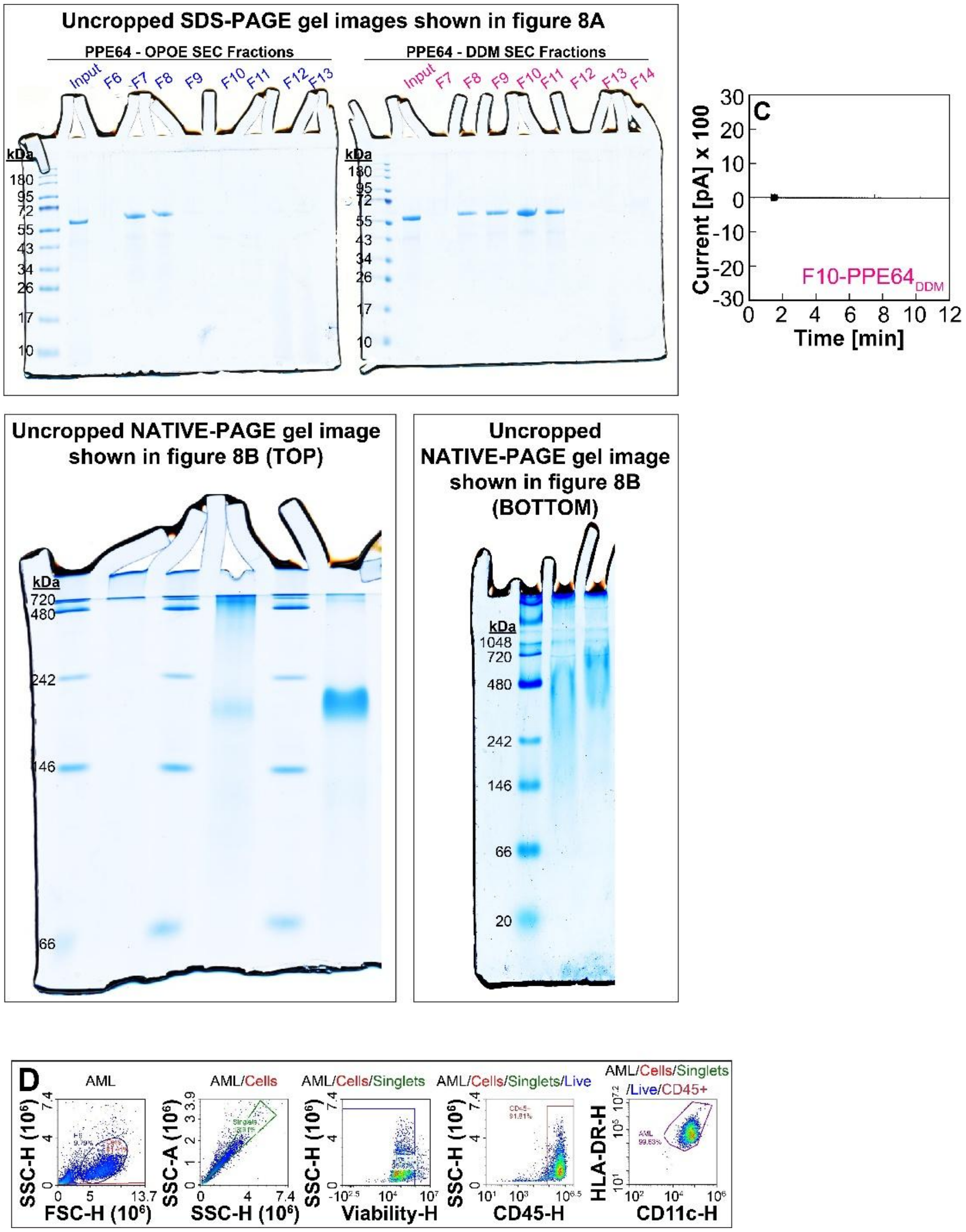

Figure S6. PPE64 protein characterization.

A-B. Uncropped images of protein gels that are shown in figure 8. **C.** Channel forming activity of PPE64-F10$_{DDM}$ in planar lipid bilayers. **D.** Generation of high purity alveolar macrophage-like cells (AMLs). Human PBMCs were differentiated into AMLs using Infasurf, M-CSF, TGF- β, and IL-10. Following 6 days of culture, cells were purified and stained with live/dead stain, CD45, and CD11c and analyzed by flow cytometry to assess purity. AMLs were approximately 99% pure following cell differentiation.

SUPPLEMENTARY TABLES

Supplementary table 1.

Strain	Parent strain and relevant genotype	Source
E. coli DH5α	*recA1; endA1; gyrA96; thi; relA1; hsdR17(rK-;mK+); supE44; ϕ80ΔlacZΔM15; ΔlacZYA-argF; UE169*	
M. tuberculosis H37Rv	wild-type	ATCC# 25618
M. tuberculosis OAL118	Δ*ppe64*::*loxP*	This study
M. tuberculosis OAL153	Δ*ppe64*::*loxP*; pOAL106; kan^R (*ppe64* integrative expression complement strain)	This study
M. tuberculosis OAL154	Δ*ppe64*::*loxP* ; pOAL103; hyg^R (*ppe64* episomal expression complement strain)	This study
M. tuberculosis OAL138	wild-type; pDM101; hyg^R (episomal expression vector strain)	This study
M. tuberculosis OAL141	Δ*ppe64*::*loxP*; pDM101; hyg^R (episomal expression vector strain)	This study
M. tuberculosis OAL151	Δ*ppe64*::*loxP* suppressor mutant	This study
M. tuberculosis OAL149	wt ; pYUB1874; kan^R (expressing heme biosensor)	This study
M. tuberculosis OAL150	Δ*ppe64*::*loxP* ; pYUB1874; kan^R (expressing heme biosensor)	This study
M. tuberculosis OAL162	Δ*ppe64*::*loxP* suppressor mutant; pOAL410; hyg^R (overexpressing *ppsA*)	This study
THP-1	Human monocytes	ATCC# TIB-202

Strains used in this work. The annotation hyg^R indicates that the strain is resistant to the antibiotics hygromycin and kan^R indicates that the strain is resistant to the antibiotics kanamycin.

Supplementary table 2.

Parent Vectors	Description	Marker
pML2424	parent vector for construction of KOs in Mycobacteria by homologues recombination; HygR	Hyg
pET21a+	plasmid used for 6His-tagged protein purification	Amp
pMN016	cloning vector for episomal expression of mycobacterial genes under strong psmyc promoter using PacI-HindIII restriction sites	Hyg
pDM101	pMN016 derivative with new multiple cloning site containing more restriction digestion sites	Hyg
pML2714	Cre recombinase vector for excision of gfp-hyg cassette utilizing loxP sites	Kan
pML2300	mycobacterial attP site integrative cloning vector for chromosomal integration and expression of genes	Kan

Gene Deletion Vectors	Description	Marker
pMLOAL104	1000bp upstream (*Spe*I-*Swa*I) & downstream (*Pac*I-*Nsi*I) of *ppe64* cloned into pML2424 (This is the knockout plasmid for deletion of *eccC4*)	Hyg

Gene Expression Vectors	Description	Marker
pDM103	*ppe64* with native promoter and RBS cloned into PacI-HindIII digested pDM101	Hyg
pDM106	*ppe64* with native promoter and RBS cloned into PacI-HindIII digested pML2300	Kan
pOAL410	*ppsA* with native RBS cloned into PacI-HindIII digested pDM101	
pYUB1874	*hs1-M7A* expression vector (gift from Dr. Amit Reddi) ref[1]	Kan

Protein purification vectors	Description	Marker
pOAL301	*ppe64* cloned into *Nde*I-*Xho*I digested pET21a+	Amp

Plasmids used in this work. The annotations hyg, kan and amp indicate resistance to the antibiotics hygromycin, kanamycin and ampicillin, respectively.

Supplementary table 3.

Gene Deletion Primers	
ppe64-KO-LF/SpeI	atatACTAGTGTTGCCAGTGAGTGGTACCC
ppe64-KO-LR/SwaI	atatATTTAAATGTTGACCTCCTCGTTACTGG
ppe64-KO-RF/PacI	aatTTAATTAACGCGACACTCACGGGTGCTG
ppe64-KO-RR/NsiI	atatATGCATTGGGCCAACTGGCATCGCAA
ppe64-V/F	GGATTGCGCATATTCTTCCG
ppe64-V/R	GCACAAGTTCCTGGACAAGA

Gene Expression Primers	
ppe64-DM101/F-PmeI	atatgtttaaacccgaccagactatcaaccaa
ppe64-DM101/R-HindIII	atatAAGCTTctaccccaacagctggcgca
ppsA-DM101/F-PacI	atatTTAATTAActtcaagcggctggacgtagccgt
ppsA-DM101/R-HindIII	atatAAGCTTtcacaccgacctctcggcct

Primers used in this work.

REFERENCES

1. Donegan RK*, et al.* Exogenously Scavenged and Endogenously Synthesized Heme Are Differentially Utilized by Mycobacterium tuberculosis. *Microbiol Spectr* **10**, e0360422 (2022).
